# Inhibition of Autophagy In Vivo Extends Methamphetamine Toxicity to Mesencephalic Cell Bodies

**DOI:** 10.3390/ph14101003

**Published:** 2021-09-29

**Authors:** Michela Ferrucci, Francesca Biagioni, Carla L. Busceti, Chiara Vidoni, Roberta Castino, Ciro Isidoro, Larisa Ryskalin, Alessandro Frati, Stefano Puglisi-Allegra, Francesco Fornai

**Affiliations:** 1 Department of Translational Research and New Technologies in Medicine and Surgery, University of Pisa, Via Roma 55, 56126 Pisa, Italy; michela.ferrucci@unipi.it (M.F.); larisa.ryskalin@unipi.it (L.R.); 2 I.R.C.C.S. Neuromed, Via Atinense 18, 86077 Isernia, Italy; francesca.biagioni@neuromed.it (F.B.); carla.busceti@neuromed.it (C.L.B.); alessandro.frati@uniroma1.it (A.F.); stefano.puglisiallegra@neuromed.it (S.P.-A.); 3 Department of Health Sciences, Università del Piemonte Orientale, Via P. Solaroli 17, 28100 Novara, Italy; chiara.vidoni@med.uniupo.it (C.V.); bcastino@yahoo.com (R.C.); ciro.isidoro@med.uniupo.it (C.I.); 4 Neurosurgery Division, Human Neurosciences Department, Sapienza University, 00135 Rome, Italy

**Keywords:** psychostimulants, stress, asparagine, glutamine, midbrain dopamine neurons, ventral tegmental area, substantia nigra pars compacta, neurotoxicity, dopamine cell loss

## Abstract

Methamphetamine (METH) is a widely abused psychostimulant and a stress-inducing compound, which leads to neurotoxicity for nigrostriatal dopamine (DA) terminals in rodents and primates including humans. In vitro studies indicate that autophagy is a strong modulator of METH toxicity. In detail, suppressing autophagy increases METH toxicity, while stimulating autophagy prevents METH-induced toxicity in cell cultures. In the present study, the role of autophagy was investigated in vivo. In the whole brain, METH alone destroys meso-striatal DA axon terminals, while fairly sparing DA cell bodies within *substantia nigra pars compacta* (SNpc). No damage to either cell bodies or axons from ventral tegmental area (VTA) is currently documented. According to the hypothesis that ongoing autophagy prevents METH-induced DA toxicity, we tested whether systemic injection of autophagy inhibitors such as asparagine (ASN, 1000 mg/Kg) or glutamine (GLN, 1000 mg/Kg), may extend METH toxicity to DA cell bodies, both within SNpc and VTA, where autophagy was found to be inhibited. When METH (5 mg/Kg × 4, 2 h apart) was administered to C57Bl/6 mice following ASN or GLN, a frank loss of cell bodies takes place within SNpc and a loss of both axons and cell bodies of VTA neurons is documented. These data indicate that, ongoing autophagy protects DA neurons and determines the refractoriness of cell bodies to METH-induced toxicity.

## 1. Introduction

Environmental stress leads to deleterious effects on the central nervous system (CNS), where brain dopamine (DA) pathways are mostly affected in both meso-cortico-limbic and meso-striatal systems [1]. Psychological stress (stress) is detrimental in DA-related psychiatric disorders and it fosters neurodegeneration [1,2,3]. Whether and how stress produces neurodegeneration of meso-striatal DA projections remains an open issue, although it is established that stress exacerbates neurotoxin-induced striatal DA depletion [4,5,6]. Striking parallels exist between stress and methamphetamine (METH) concerning both neurochemistry and neurotoxicity, which witnesses for a deleterious overlapping and synergism between molecular mechanisms operating following both stimuli [7,8,9,10,11,12,13]. This explains translational relevance of psychostimulants and stressful agents, which converge on DA transmission [14,15,16,17,18].

In line with this, some neuroprotective strategies such as autophagy may be effective to counteract both psychological stress- and psychostimulant-induced toxicity to DA neurons [1,19,20,21,22]. In fact, both psychological stress and psychostimulants target DA transmission both within the meso-striatal and meso-limbic pathways [1]. Therefore, a deeper knowledge on how autophagy modulates METH toxicity in vivo is supposed to translate beyond the pathology induced by psychostimulants to encompass neurodegeneration following stressful events. Thus, studying the role of autophagy in METH-induced brain damage in vivo is relevant nowadays in various fields of neuroscience.

Therefore, in the present study the effects of the psychostimulant METH alone or in the presence of two autophagy inhibitors, are investigated in vivo on mesencephalic DA systems. METH alone is well-known to induce neurotoxicity for meso-striatal DA terminals in vivo, which was early described in primates [23], then reproduced in rodents [24,25] and finally demonstrated in human abusers [26,27]. Such a model was selected here since METH per se produces an impairment of the autophagy flux in vitro and in vivo [15,21,28,29,30]. When this present experimental work was early planned some involvement of autophagy in METH toxicity was already established in vitro [15,28] and in vivo [29,31]. Despite evidence indicated that METH administration strongly engages autophagy, it was unclear whether autophagy was either detrimental or beneficial for METH toxicity. Further in vitro studies clarified such an issue by showing that stimulation of autophagy during METH administration does produce a robust protective effect [22]. In fact, when METH is administered to cell cultures in the presence of either pharmacological or genetic autophagy inhibitors, METH toxicity is worsened [22]. In contrast, when autophagy is stimulated by inhibiting the mechanistic Target of Rapamycin (mTOR) METH toxicity is prevented [30]. This evidence derives from in vitro studies, which need to be validated in vivo, where METH-induced effects within an intact CNS are way more complex.

In fact, in vivo midbrain DA cell bodies are refractory to METH toxicity, which is unique compared with other DA neurotoxins. This is likely to rely on the molecular mechanisms, which specifically characterize METH toxicity in vivo, such as a massive DA release, which is produced by METH administration [8]. This in turn, produces METH-induced DA self-oxidation, which in turn alters cysteinyl groups within amino-acid chains, thus producing protein misfolding [32,33,34]. This is the trigger to recruit autophagy as a compensatory mechanism to remove misfolded proteins up to a level, which exceeds its capacity [22].

In the present study, we investigate the role of autophagy on METH toxicity in vivo. Since METH destroys meso-striatal DA axon terminals, while fairly sparing mesencephalic cell bodies, in the present work we specifically question whether such a refractoriness of mesencephalic DA cell bodies to METH toxicity could be disrupted by impairing the autophagy machinery.

With this aim, we challenged the mesencephalic DA system with moderate doses of METH in combination with systemic autophagy inhibitors (either asparagine, ASN or glutamine, GLN) to test whether toxicity for DA cell bodies could be frankly induced by METH+ASN (or GLN). Additionally, we explored whether autophagy inhibition may extend METH toxicity to other midbrain DA nuclei, beyond Substantia Nigra pars compacta (SNpc), such as the Ventral Tegmental Area (VTA). Thus, we administered C57Bl/6 the amino acids ASN or GLN, which are known to inhibit the autophagy pathway [35,36,37,38,39,40,41,42,43,44,45], in combination with slight doses of METH. The effects of ASN/GLN administered systemically on the autophagy status of mesencephalic DA cell bodies were assessed in parallel experiments aimed to establish whether this experimental procedure impairs indeed the autophagy pathway within the midbrain tegmentum, at the time when METH is administered. The present experiments were analyzed by counting mesencephalic neurons according to a stereology-like procedure being validated by a number of studies [46]. Such a flexible stereology allows to count neurons owing a significant cell body volume variability, thus applying to various zones of the SNpc (i.e., ventral and dorsal tier) as well as to neighboring neurons within VTA. This experimental protocol allows to address the question of whether refractoriness of SNpc cell bodies and whole VTA neurons (both cell bodies and axon terminals) to METH depends on an effective autophagy machinery.

## 2. Results

### 2.1. ASN (or GLN) Suppresses Autophagy and Induces Bax Overexpression When Combined with METH

**Figure 1:**
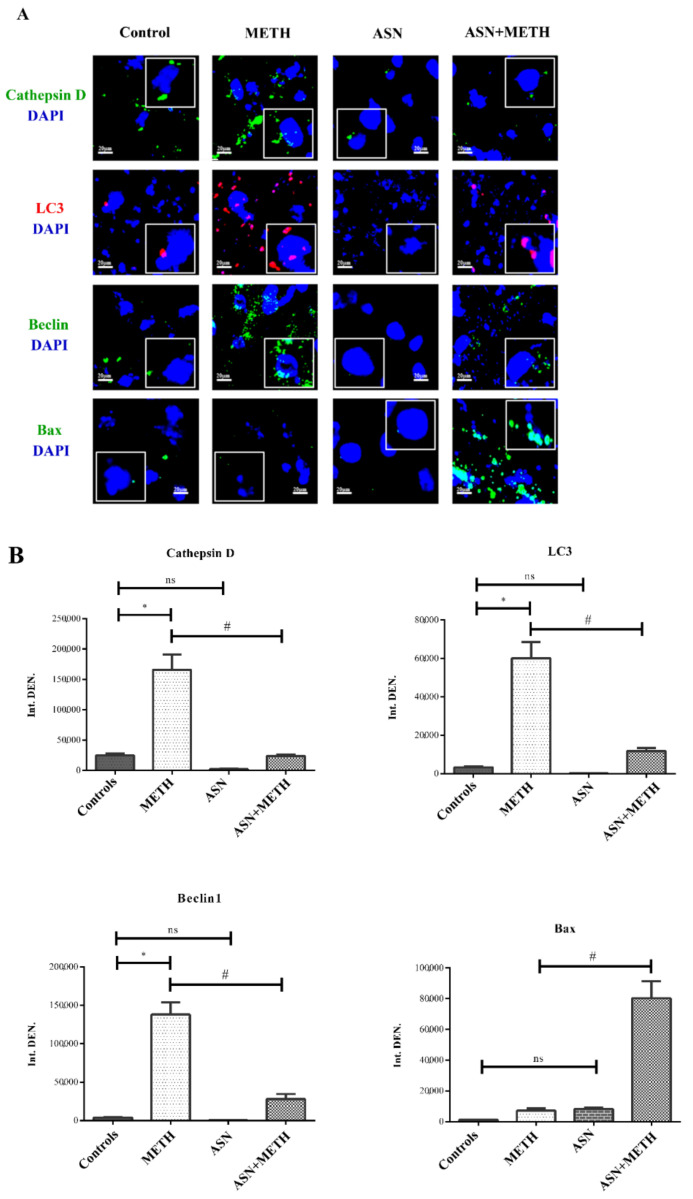
ASN impairs autophagy and worsens METH toxicity. (**A**) Representative pictures from midbrain tegmentum showing immunofluorescence for autophagy-related proteins such as LC3II (the lipidated isoform of the protein hallmark protein of autophagosomal membrane), Beclin1 (an interactor of PI3KC3, which promotes autophagosome biogenesis) and Cathepsin-D (a lysosomal protease). In addition, immunofluorescence for the apoptosis-related antigen Bax is shown representatively. Pictures are obtained from mice administered either saline, METH, ASN and the combination ASN + METH. The insert magnifies specific puncta (or fluorescent aggregates). Arrows indicate each specific antigen. (**B**) Graphs report densitometry for each protein. Data are given as Arbitrary Units ± S.D. and represent the average IntDEN of five microscopic fields randomly chosen (IntDEN = integrated density is the product of Area of selected cell X Mean fluorescence calculated with the software ImageJ). ns = not significant. * *p* < 0.05 compared with Controls; ^#^ *p* < 0.05 compared with METH. Scale bar = 20 μm.

**Figure 2:**
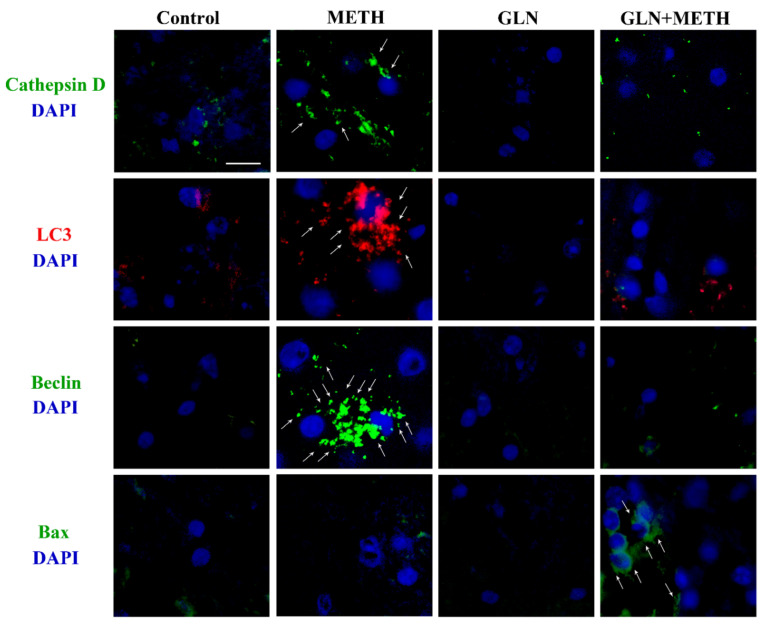
GLN impairs autophagy and worsens METH toxicity. Representative pictures from midbrain tegmentum showing immunofluorescence for the very same autophagy-related proteins LC3II, Beclin1 and Cathepsin-D and the apoptosis-related antigen Bax shown in Figure 1, in mice treated with saline (Control), METH, GLN and combined METH and GLN. Arrows indicate each specific antigen. Scale bar = 25 μm.

Immunofluorescence within midbrain tegmentum for autophagy-related proteins such as LC3, Beclin1 and Cathepsin-D increases following METH (Figure 1A,B and Figure 2). In contrast, ASN (1000 mg/kg) or GLN (1000 mg/kg) depress baseline and METH-induced increase of the very same proteins, as shown in representative Figure 1A and Figure 2 and measured in graphs of Figure 1B and Table 1. In detail, both ASN and GLN alone depress both LC3 and Beclin1 immunofluorescence compared with control. In contrast, this immunofluorescence increases in mice administered METH alone (Figure 1A,B and Figure 2 and Table 1). When combined administrations are carried out, ASN or GLN occlude the effects of METH. This is replicated when Cathepsin D is stained. Altogether, these data validate ASN or GLN as experimental tools to inhibit autophagy within DA cell bodies of the mesencephalic tegmentum. In order to assess the amount of LC3 within the mesencephalon, the immunofluorescent area per cell was calculated from neurons of the ventral tier of the SNpc, which feature a highly homogeneous cell size and shape, with a negligeable variability in cell size (S.E.M. less than 1%, as reported in the Section 4.

In detail, as shown in Table 1, when the mean LC3 immunofluorescent area/cell is calculated, ASN alone decreases LC3 immunofluorescence more than GLN alone both compared with their own controls (27.11% ± 1.26% and 56.56% ± 13.94%, respectively, considering control = 100%). METH alone massively increases LC3 immunofluorescent area/cell as reported in Table 1. As expected, both combined treatments ASN + METH or GLN + METH decrease the amount of METH-induced overexpression of LC3 immunolfuorescent area (37.67% ± 7.54% for ASN and 25.19% ± 2.82% for GLN, compared with METH). This corresponds to a decrease in LC3 immunofluorescent area/cell for ASN and GLN of 2.65-fold and 3.97-fold, compared with METH, respectively. Despite similar effects are obtained for ASN and GLN both alone and following their combination with METH, there are slight discrepancies between the quantitative effects produced by these amino-acids which are likely to depend on slight differences in their molecular mechanisms as autophagy inhibitors [35,36,37,38,39,40,41,42,43,44,45].

When ASN or GLN are administered in combination with METH, the apoptosis-related antigen Bax becomes strikingly evident. This latter finding provides preliminary evidence for the apoptotic nature of METH-induced cell death, when autophagy is occluded.

### 2.2. ASN (or GLN)-Induced Autophagy Inhibition Dramatically Worsens METH-Induced Toxicity to Meso-Striatal DA Axons

**Figure 3:**
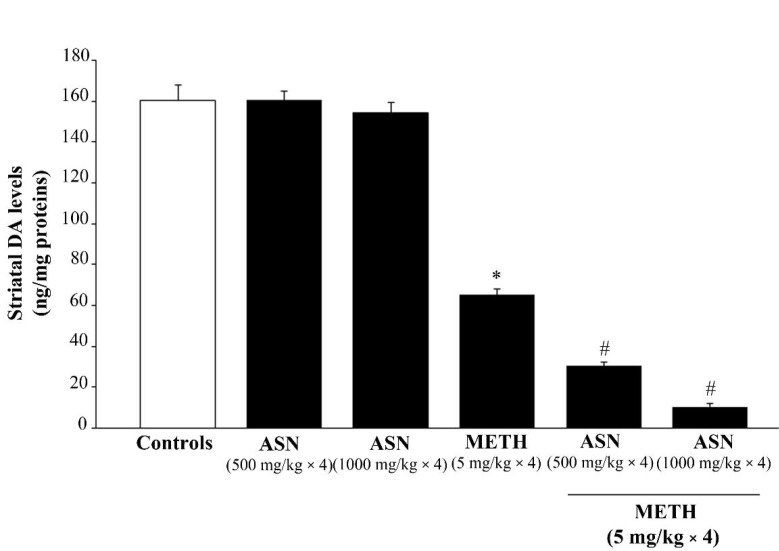
ASN administration dose-dependently exacerbates METH-induced striatal DA loss. Graph reports DA levels measured by reversed phase HPLC coulometric detection of striatal homogenates following ASN and/or METH administration. * *p* < 0.05 compared with Controls; ^#^ *p* < 0.05 compared with METH.

ASN administration per se does not modify striatal DA levels compared with controls at any dose (Figure 3). In contrast, METH administration produces over 60% of striatal DA depletion compared with controls (Figure 3). In mice receiving combined treatment, ASN exacerbates dose-dependently METH-induced striatal DA toxicity (Figure 3). In fact, in mice receiving ASN at the lower dose (500 mg/kg) combined with METH, striatal DA levels are 18.89% of controls and 46.49% of that measured in mice receiving METH alone (Figure 3). When mice were administered the higher dose of ASN (1000 mg/kg) before METH, striatal DA levels are suppressed to 6.59% of controls and 16.21% of those from mice treated with METH alone (Figure 3).

**Figure 4:**
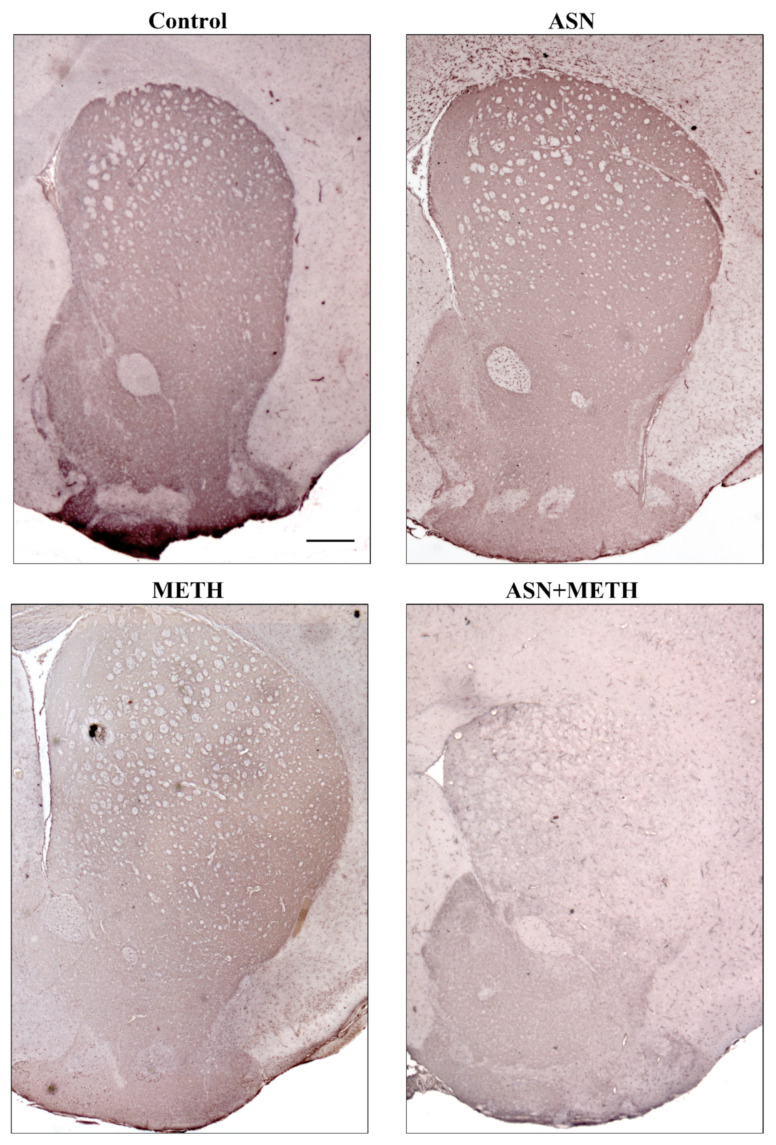
ASN administration worsens METH-induced striatal loss of TH immunostaining. Representative pictures of TH immunohistochemistry within the striatum in a Control mouse and a mouse administered either ASN (1000 mg/kg × 4), or METH (5 mg/kg × 4), or combined ASN (1000 mg/kg × 4) + METH (5 mg/kg × 4). Scale bar = 380 μm.

Representative striatal immunohistochemistry for the rate-limiting catecholamine-synthesizing enzyme tyrosine hydroxylase (TH, Figure 4) and densitometric analysis (Figure S1A, Supplementary Materials) indicate that, ASN (1000 mg/kg) while not affecting TH immunostaining when administered alone, enhances METH-induced loss of TH immunostaining (Figure 4). Similar results were obtained when GLN was administered as an autophagy inhibitor (Figure S2, Supplementary Materials). Altogether, these data indicate that in vivo ASN (or GLN) administration, while inhibiting autophagy within mesencephalon, exacerbates meso-striatal DA axonal loss induced by METH.

### 2.3. Inhibition of Autophagy by ASN (or GLN) Extends METH Toxicity to Midbrain DA Cell Bodies

**Figure 5:**
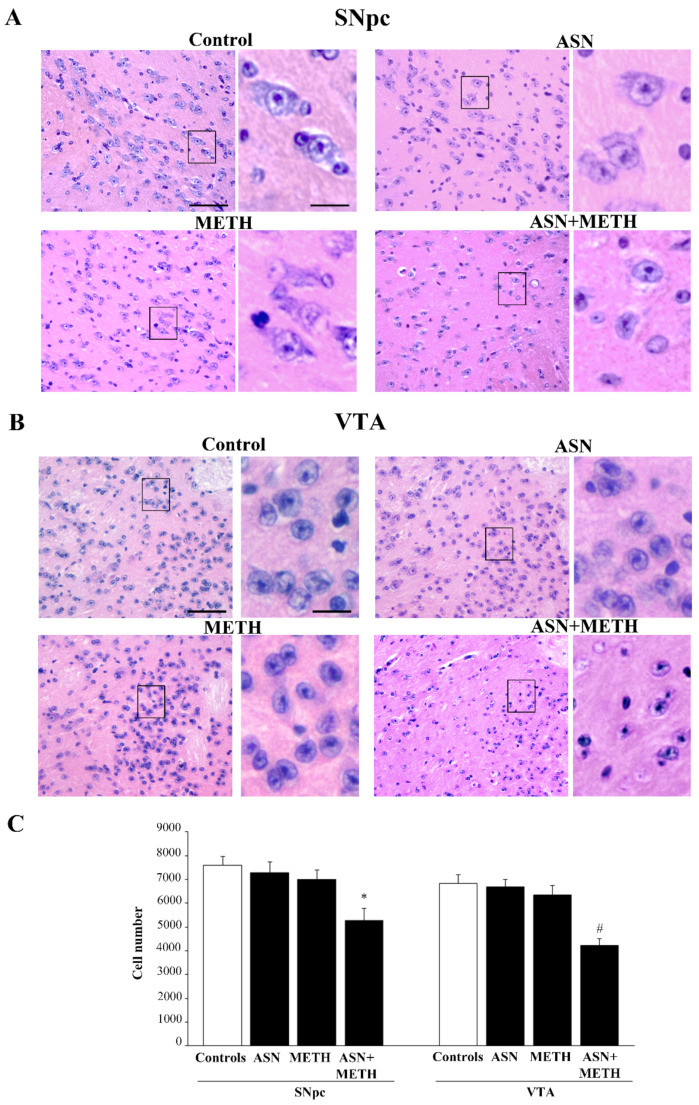
Combined ASN + METH reduces the number of cell bodies within both SNpc and VTA. Combined treatment ASN + METH reduces the number of cells within SNpc and VTA. Representative H&E-stained pictures from SNpc (**A**) and VTA (**B**). Images at high magnification refer to black-squared areas of the related low magnification pictures. (**C**) The histogram reports the number of H&E-stained cells counted within both areas according to stereology-like procedure. * *p* < 0.05 compared with Controls; ^#^ *p* < 0.05 compared with Controls and METH. Scale bar (low magnification) = 80 μm; scale bar (high magnification) = 18 μm.

**Figure 6:**
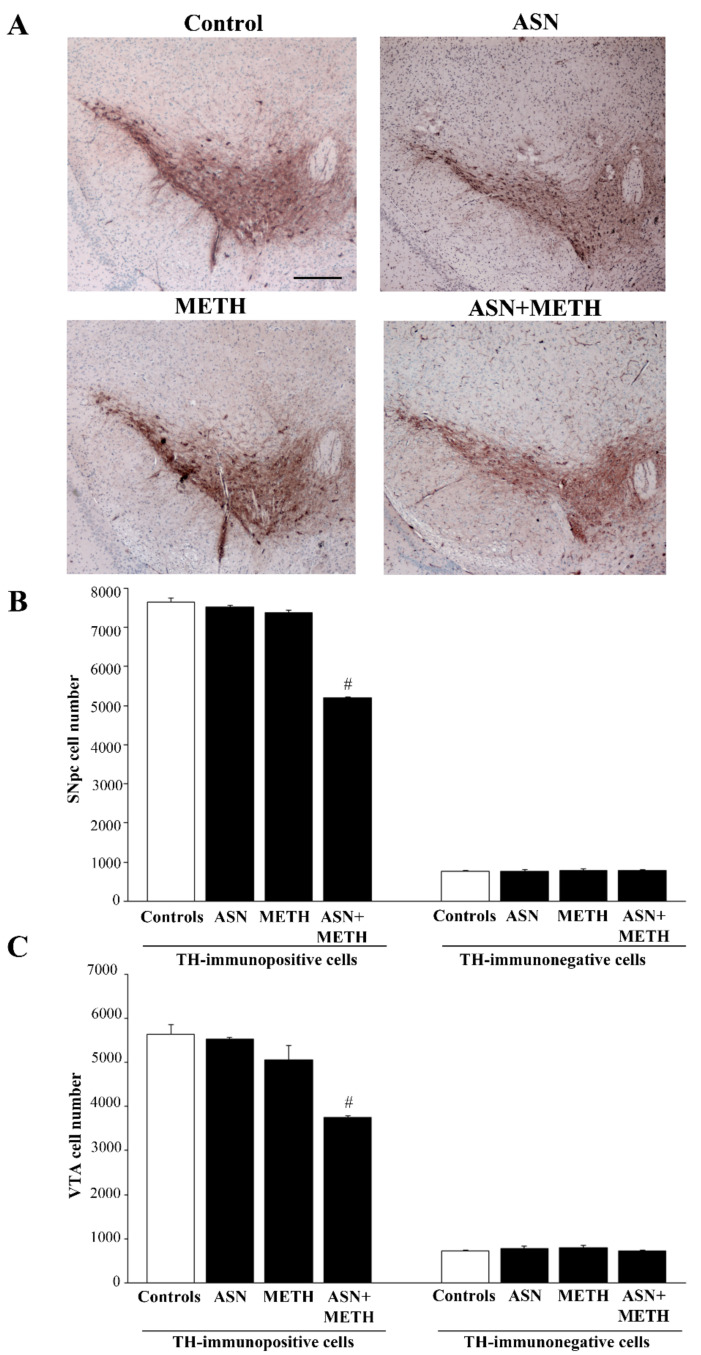
ASN extends METH toxicity to cell bodies within SNpc and VTA. (**A**) Representative pictures of TH immunostaining within the midbrain from a control mouse and from mice treated with ASN (1000 mg/kg × 4), or METH (5 mg/kg × 4, 2 h apart), or combined ASN (1000 mg/kg × 4) + METH (5 mg/kg × 4, 2 h apart). The stereology-like count of TH-immunopositive and TH-immunonegative cells within SNpc (**B**) and VTA (**C**) following various treatments is reported. ^#^ *p* < 0.05 compared with Controls and METH. Scale bar = 200 μm.

**Figure 7:**
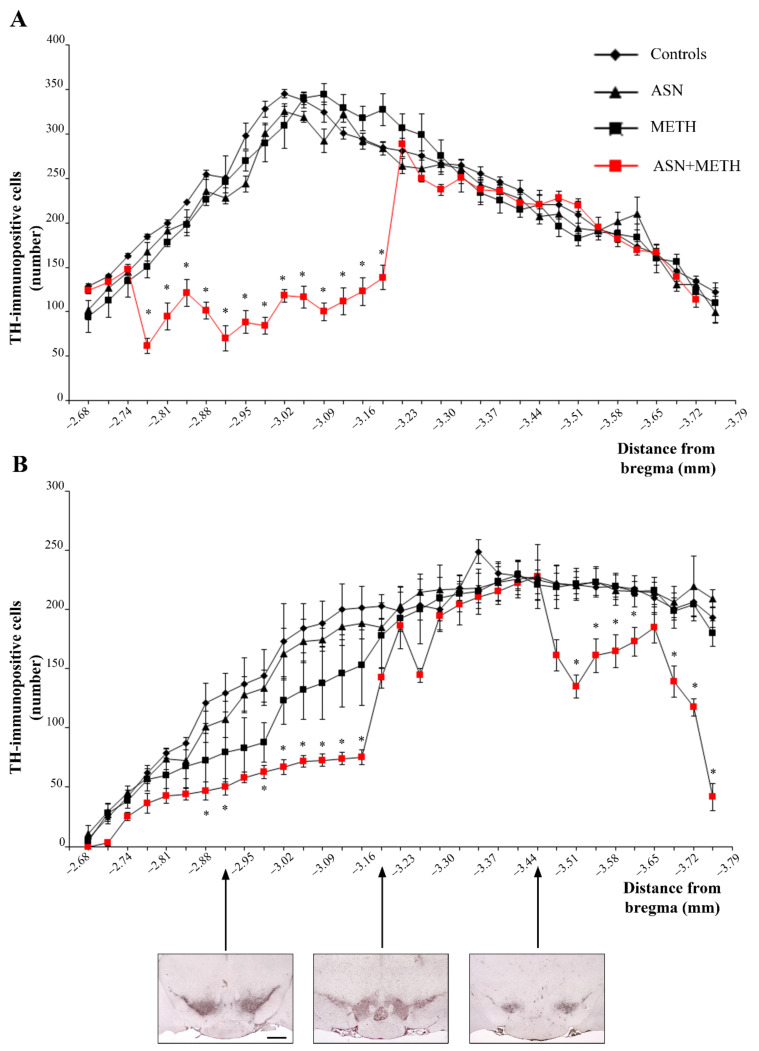
Counts of TH-immunopositive cells within SNpc and VTA following ASN and METH administration. Graphs report the number of TH immunopositive cell bodies which were counted within each slice in (**A**) SNpc and (**B**) VTA. Counts are carried out across the whole rostro-caudal extent of both nuclei. Representative images from three mesencephalic levels are reported in the bottom part of the figure. Each level is identified through its stereotaxic coordinates. * *p* < 0.05 compared with Controls. Scale bar = 480 μm.

**Figure 8:**
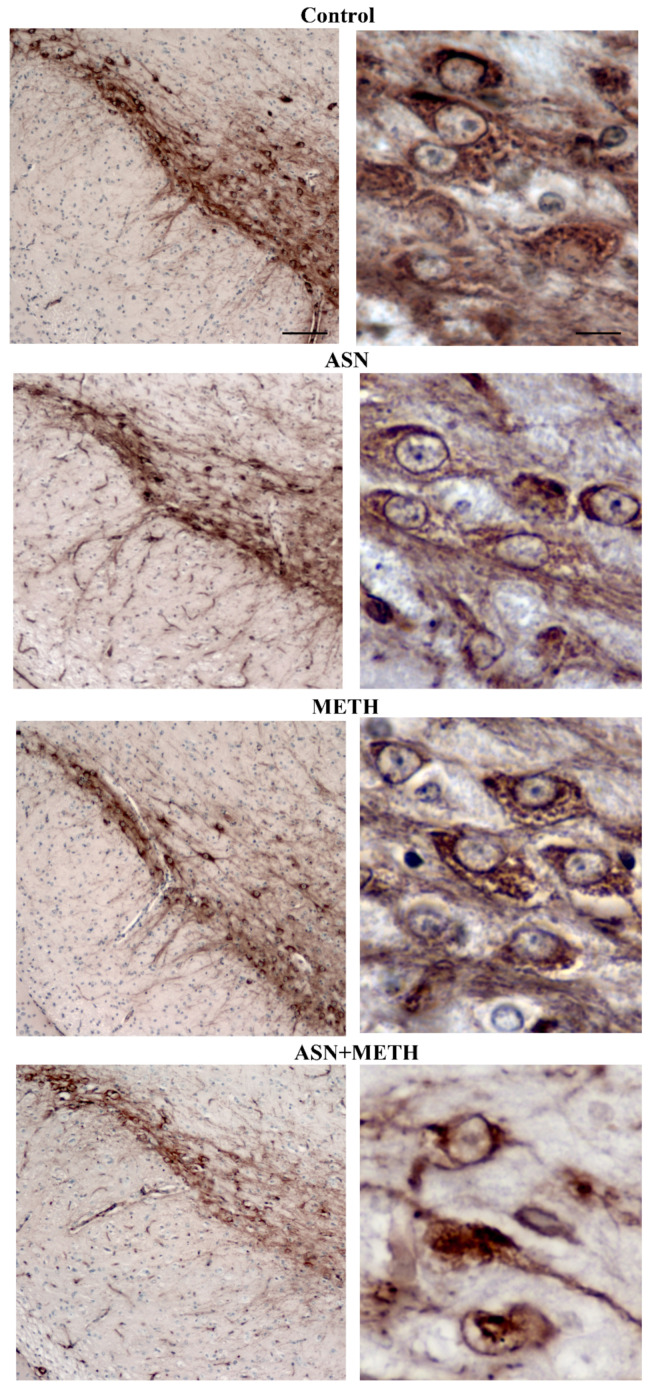
Representative pictures of TH-immunopositive cell bodies within SNpc. Combined treatment with ASN and METH alters the cell body of TH-immunopositive neurons within SNpc. Scale bar (low magnification) = 100 μm; scale bar (high magnification) = 12 μm.

**Figure 9:**
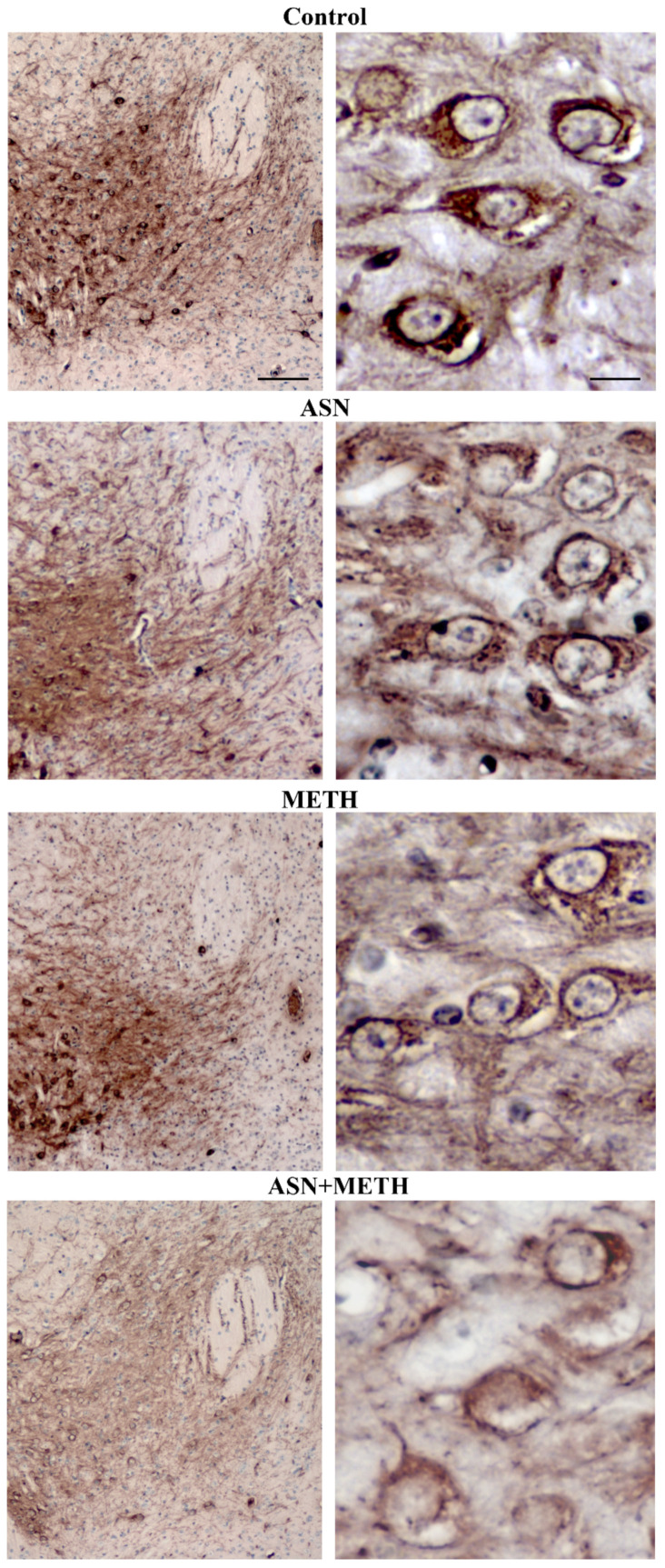
Representative pictures of TH-immunopositive cell bodies within VTA. Combined treatment with ASN and METH affects the cell body of TH-immunopositive neurons within VTA. Scale bar (low magnification) = 100 μm; scale bar (high magnification) = 12 μm.

In order to investigate whether an autophagy impairment induced by ASN may extend to midbrain DA cell bodies, we applied a stereology-like count. In detail, the effects of various treatments on DA neuronal cell bodies, which project to the dorsal striatum, the *nucleus accumbens*, the olfactory tubercle, the isocortex and limbic allocortical areas such as the piriform cortex, were assessed by H&E histochemistry and TH immunohistochemistry. The staining was carried out at the level of midbrain DA nuclei (Figure 5, Figure 6, Figure 7, Figure 8 and Figure 9). Serial 7-μm thick slices, spaced 35-μm, were collected and used for cell counting by using a stereology-like procedure after either H&E staining (Figure 5) or TH immunoperoxidase (Figure 6). As shown following H&E staining, in mice treated with ASN or METH alone cell density was similar to a saline-administered Control mouse (Figure 5A,B). In contrast, a decrease in the number of cell bodies is shown in a mouse receiving combined ASN + METH administration (Figure 5A,B). Cell count indicates that ASN + METH dramatically reduces midbrain cell bodies within both SNpc and VTA (Figure 5C).

It is remarkable that only a combined administration of ASN + METH produces cell loss, while this never occurs either in the midbrain from mice treated with ASN alone, or from mice administered METH alone. In fact, in the present experimental conditions METH alone never produces cell loss (Figure 5). The specific quantitative evaluation of nigral cell bodies was carried out by counting TH-immunopositive and TH-immunonegative cells separately (Figure 6). This allows to demonstrate that combined ASN + METH administration selectively involves TH-immunopositive cells both within SNpc and VTA (Figure 6B,C and Figure 7). Similar results were obtained in SNpc and VTA when GLN was administered as an autophagy inhibitor (Figure S3, Supplementary Materials).

Cell loss is not due to a loss of expression of the TH protein since it is confirmed by using H&E. Spared cells following combined treatment ASN + METH are often altered with cytopathological features such as an altered neuronal shape, H&E-staining density with pale cytosolic areas and picnotic nuclei and reduced cell size (Figure 8 and Figure 9). Remarkably, spared cells express a lower amount of TH (Figure 8 and Figure 9, high magnification and Figure S1B,C). A remarkable damage is documented in the rostral half of the SNpc compared with its caudal extent (Figure 7A). Within VTA, the middle rostro-caudal extent is almost spared, while cell loss is significant towards the rostral and caudal poles of the nucleus (Figure 7B). It is worth noting that ASN alone produces an altered cell morphology compared with controls consisting in faint eosin staining, which is likely to be due to large empty vacuolar compartments, as previously described at TEM, where a decreased cytosolic electrondensity was shown [30].

**Figure 10:**
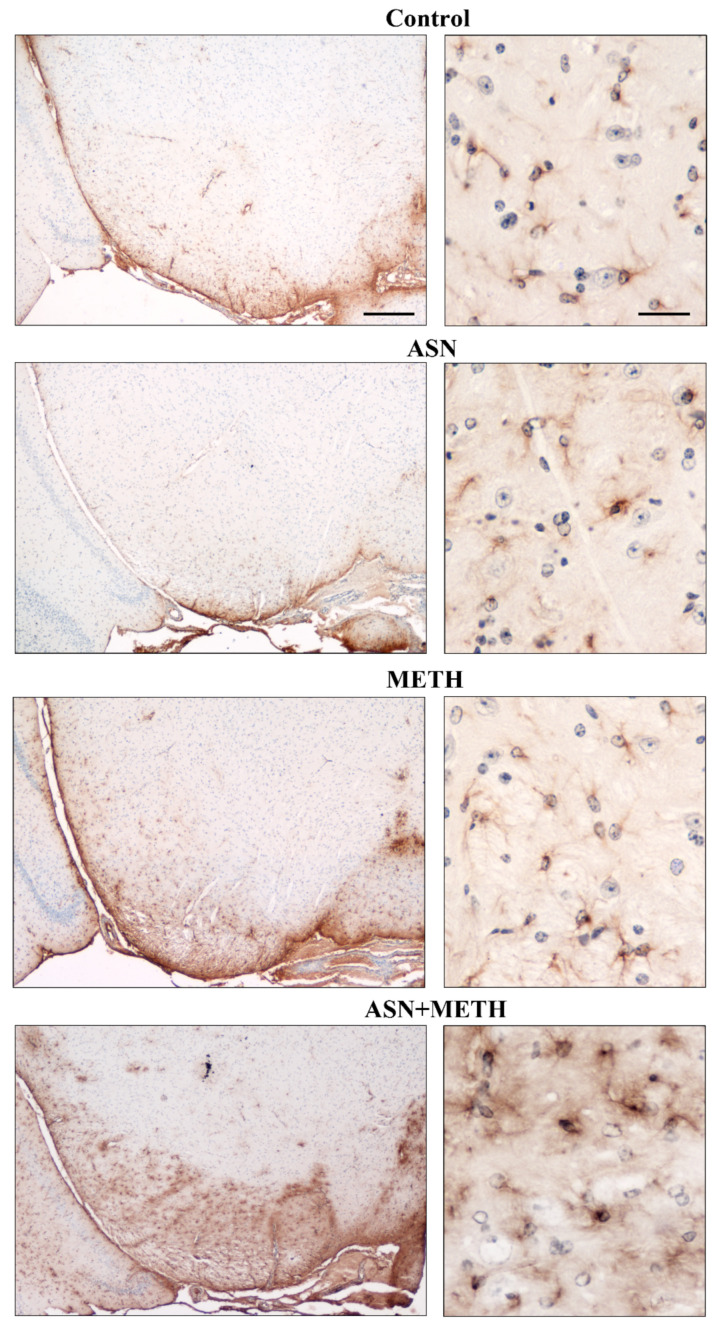
Representative pictures of GFAP immunostaining within SNpc and VTA. Combined treatment with ASN + METH increases GFAP immunostaining within SNpc and VTA. Scale bar (low magnification) = 250 μm; scale bar (high magnification) = 40 μm.

Remarkably, the decrease of TH immunohistochemistry within SNpc and VTA, which occurs following combined ASN + METH-administration is accompanied by an increase in glial fibrillary acidic protein (GFAP) immunostaining (representative pictures of Figure 10 and densitometric analysis reported in Figure S1D). Such an increase in GFAP, which typically occurs in reactive astrocytes during neuronal damage, is in line with the loss of neuronal cell bodies measured within the midbrain tegmentum from ASN + METH treated mice.

## 3. Discussion

In the present manuscript, we provide evidence that the psychostimulant METH, when administered alone at the dose of 5 mg/kg × 4, 2 h apart, significantly reduces striatal DA levels, which is concomitant with a decrease in striatal TH immunostaining. These findings are consistent with previous literature, which indicates that in vivo METH produces a damage to DA neurons, which is limited to axon terminals and it is confined to the meso-striatal pathway [8,47,48]. In these experimental conditions, when METH is administered following autophagy inhibitors such as ASN or GLN, METH toxicity is worsened. In detail, ASN or GLN, at the dose of 1000 mg/kg, while impairing the autophagy machinery within mesencephalic cell bodies, converts METH toxicity from a selective damage to striatal DA terminals into a frank cell body loss within SNpc. Additionally, both axons and cell bodies of VTA neurons are damaged following combined treatments. This extends METH toxicity to the whole DA neuronal compartments of the mesencephalon. Remarkably, METH-induced damage to the cell bodies under concomitant ASN or GLN + METH administration extends medially to the tegmentum of the cerebral peduncle, thus recruiting DA neurons within the VTA of Tsai. This finding was never described so far. In these experimental conditions, the loss of striatal axon terminals measured as a decrease in DA levels within striatal homogenates and a decrease in striatal TH immunohistochemistry worsens, as well. This suggests that ASN does not merely enhance METH toxicity, but it rather modifies the kind of DA toxicity extending the damage to all mesencephalic DA nuclei. A concomitant loss of cell bodies within SNpc and VTA following METH was never described so far. This suggests that autophagy status is key to tune the vulnerability of DA neurons to psychostimulants and, possibly, stressful conditions.

The present study leads to hypothesize an autophagy-dependent site-specificity for METH-induced neurotoxicity. This determines the specific compartmentalization (axons vs. cell bodies) of METH-induced neurotoxicity. This is consistent with the fact that, within axon terminals, METH-induced DA release and oxidation is massive [31,49,50,51,52,53], which presumably overwhelms at large the buffering effects of endogenous autophagy. In contrast, the DA release at the level of mesencephalic cell bodies is much lower [54,55,56], which makes baseline autophagy sufficient to effectively counteract METH-induced toxic effects. This may explain why, when suppressing autophagy in vivo, a frank damage to nigral cell bodies occurs, even following moderate doses of METH. Most studies using METH, even at high doses, across various animal species, despite documenting consistently a loss of meso-striatal DA terminals, reported only some, if any, loss of nigral cell bodies. On this basis, here, we hypothesize that, the pattern of METH-induced DA neurotoxicity is restricted to axon terminals, since mesencephalic DA cell bodies are protected by the endogenously ongoing autophagy activity. The present experiments address this hypothesis by analyzing in vivo METH toxicity in the presence of both ASN and GLN as systemic autophagy inhibitors.

In previous studies from our group, a loss of DA cell bodies was never obtained in this mouse strain at any age (ranging from 8 weeks up to 24 weeks) and in any housing condition or environmental temperature. When counting cell bodies at nigral level following METH alone, by using stereology-like procedure, we failed to document a loss of neurons in the mesencephalon, both in the A9 area (i.e., the SNpc) and the close A10 area (i.e., the VTA). Nonetheless, in recent years some reports [57,58,59] by using extreme approaches documented some cell loss, only within SNpc, which occurs when METH is administered at high doses. Roughly, cell loss in the SNpc is described when the loss of striatal DA levels exceeds 90% and the amount of striatal DA innervation decreases by roughly 90%. Thus, METH toxicity in vivo, in rodents and primates, despite being traditionally described as a pure axonal toxicity, may also involve cell bodies when administered in very high amounts. Most studies failed to document a loss of cell bodies, even considering that, when robust doses are administered a high lethality occurs. It is likely that, different results by using high amount of METH may rely on the high variability of METH toxicity, depending on the temperature, housing conditions, including the number of animals per cage, the food and other items (the species, the strain and the gender and even the purchaser of the rodent) [25,60,61].

The loss of striatal DA axon terminals reflects an authentic toxicity, which is restricted to the axonal compartment. This is not the result of a synaptic remodeling since DA axons degenerate as confirmed directly by cupric-silver staining and TH immunostaining, which is a gold standard procedure to document an actual toxicity to meso-striatal DA axons [59]. As stated previously, for several decades no damage could be described at the level of the SNpc concerning DA cell bodies.

What might be the specific reason, inherent to the molecular mechanisms of METH toxicity, which makes the cell bodies refractory compared with distal axons?

It is well-known that METH-induced toxicity associates with autophagy impairment [22,29], we postulated here that the autophagy machinery is key in vivo to protect DA cell bodies compared with DA axons against METH toxicity. This is likely to be due to the preferential effects of METH in disrupting DA storage within vesicles. The massive release of DA within cytosol is supposed to exceed several-fold what happens within axoplasm, considering the low DA levels in the cell body. Nigral DA mostly occurs within recurrent axon collaterals, which may produce a moderate stress to nigral cell bodies. The presence of collaterals in the meso-striatal system is typical while it does not occur for DA neurons placed in the VTA [62], which project to the meso-limbic and meso-cortical pathways. Such a difference in the amount of extracellular nigral DA, provided by axon collaterals within the SNpc compared with VTA, may further explain the intrinsic refractoriness of VTA compared with SNpc neurons to METH toxicity.

Why DA plays a major role in producing METH toxicity?

As demonstrated in the pioneer study by Schmidt et al. (1985) [24]. When studying the mechanisms of METH toxicity, it is known that METH produces a massive increase in the amount of freely diffusible DA [8,31,63,64]. This effect is achieved by METH through its gateway into the DA axons, mainly occurring via the DA transporter (DAT) [65,66,67], even though it was recently shown that a passive diffusion of METH across the plasma membrane may also occur [68]; thus, the blockade of DAT does not completely occlude METH toxicity. In any case, once in the DA terminals, METH is able to revert DAT [65,69] and it mostly interacts with the neurotransmitter vesicles, which are present at the level of the axon terminals [66]. At this level, METH alters the activity of the vesicular monoamine transporter-2 (VMAT-2) both blocking its highly selective uptake of cytosolic DA into vesicles [70] and by misplacing the molecular complex VMAT-2 moving it from the vesicles towards stochastic axoplasmic compartments, such as the trans-Golgi network and other vesicular components, but the neurotransmitter vesicles [71,72,73]. In addition, when present in the vesicle, METH blocks the proton gradient by impairing the proton import pump, which produces the acidification of the vesicle [28]. In baseline conditions, the acidification of the vesicle is produced by importing H^+^ ions within the vesicle. This produces a decrease of the pH by roughly 3 logarithmic units, which means an increase of 1,000 H^+^ ions. Thus, the pH of the vesicle roughly corresponds to 4.2. The presence of METH counteracts such a process and rises the vesicle pH up to 7.2, which is similar to the pH measured within surrounding cytosol in the DA axon. In this way, DA, which is a weak base, once within the vesicles, within an acidic compartment, behaves as an authentic basic compound and it is polarized by binding H^+^ ions, thus leading to a positive charge. In this way, most vesicular DA is a polar cation, which cannot freely diffuse through the lipophilic vesicle membrane. This mechanism grants the physiological DA vesicle storage. This is disrupted in the presence of METH, which suppresses vesicle acidification [74,75]. This renders non-polar most DA molecules, which freely diffuse and spread in the axoplasm [28]. This occurs suddenly and massively, thus creating high amount of freely diffusible DA, which invades the axoplasm. This occurs concomitantly with the reversible inhibition of the monoamine oxidase type A (MAO-A) [76,77] placed on the mitochondrial outer membrane within the DA terminal. Thus, METH accelerates a toxic interaction between highly reactive DA, which self-oxidizes into DA quinones and other oxidative species to oxidize cysteinyl groups to misfold a variety of proteins [33,34,78,79]. Among these proteins, alpha-synuclein plays an important role [22,29]. Alpha-synuclein may buffer METH toxicity by binding such oxidized DA forms, as shown by Schlüter et al. (2003) [80]. In this study, METH administration to alpha-synuclein knock-out mice produced a more severe toxicity. The high amount of protein oxidation, which is produced by METH and directly by DA under the effects of METH, produces a high amount of misfolded proteins and dysfunctional mitochondria, which turn to overwhelm the clearance reservoir of the neuron.

The autophagy machinery plays a seminal role in clearing a variety of misfolded proteins. In fact, it plays an important role in protecting from METH toxicity [22,30]. As expected by the overwhelming amount of cargoes produced by METH, autophagy is perturbed and may fail to clear the cell. A failure in the autophagy machinery is expected to impede the clearance of misfolded proteins, while it inhibits mitochondriogenesis and mitochondrial removal [30], which explains why during METH toxicity, autophagy impairment enhances the amount of misfolded proteins and altered mitochondria.

The autophagy perturbation under the effect of METH was firstly described by Cubells et al. (1994) [28]. This was confirmed later by Larsen et al. (2002) [15]. Recent studies indicate a specific autophagy defect [22], where METH alters the effectiveness of autophagosomes [30]. Thus, under the effects of METH autophagy inducers protect while autophagy inhibitors worsen METH toxicity. This evidence so far was produced in vitro by Castino et al. (2008) [22], who demonstrated that under the effect of 3-methyladenine (3MA) or by using a mutant form of the VPS34, which in both cases occlude autophagy, there is a marked impairment in the autophagy machinery. This is evident by the accumulation of specific autophagy markers and substrates such as α-synuclein, which cannot be removed by the autophagy flux. In such a condition of autophagy impairment, a massive activation of the caspase takes place, which leads to cell death. A similar result was recently obtained by administering in vitro the autophagy inhibitor ASN which produced an increase of altered mitochondria and misfolded proteins in vitro [30]. Contrariwise, the autophagy activator rapamycin, when administered in vitro powerfully protects against METH toxicity with a suppression in the amount of misfolded proteins and a burst of mitochondrial biogenesis [30]. In detail, very recent studies demonstrate the specificity of the blockade of the autophagy flux under the effects of METH [30]. This is not due to a mere lack of fusion between autophagosomes with lysosomes. It is rather the commitment of autophagosomes, which is impaired under the effects of METH. Lazzeri et al. 2018 [30], showed that autophagosomes are neutralized in their clearing activity of proteins and mitochondria by METH. This is due to the dispersion of the autophagy-specific protein LC3 from autophagy vacuoles and mitochondria towards the cytosol.

## 4. Materials and Methods

### 4.1. Animals

C57Bl/6 male mice (N = 108, Charles River Calco, Milano, Italy) were housed in small cages (*n* = 3 per cage; cage length = 25 cm; cage width = 20 cm; cage height = 13 cm) and kept under controlled environmental conditions (temperature = 22 °C; humidity = 40%; 12 h light/dark cycle) with food and water ad libitum. All these measures were kept constant all over the study since the effects of METH markedly vary depending on housing conditions [24]. These mice were divided into different groups of treatment and administered with METH and ASN (see Section 4.2).

Additional experiments were carried out in a total of N = 32 C57Bl/6 male mice (Charles River, Milano, Italy). These mice were divided into different groups of treatment and administered with METH and GLN (Sigma-Aldrich, St. Luis, MO, USA) (see Supplementary Materials).

The mouse strain C57Bl/6 was chosen due to a very stable DA phenotype and the highest sensitivity to DA neurotoxins, as ascertained by previous studies [25,29,80,81,82]. This allows to better elicit DA toxicity under appropriate experimental conditions.

The present experiments were never submitted as a manuscript to any journal. These data were partially communicated in previous meetings by F.F and M.F. [83,84]. Ethics in experiments followed the Guidelines of the European Council (86/609/EEC) and concerning the specific procedures here reported in full adherence with the NIH guide for the use and care of Experimental Animals, which represented the gold standard reference for the use and care of laboratory animals. The experiments were approved by the local Ethical Committee.

### 4.2. Treatments and Experimental Design

METH hydro-chloride was kindly gifted by Medicina Legale (Forensic Medicine Institute, at the University of Pisa, Professor Mario Giusiani). METH was dissolved in saline and it was administered i.p. at the dose of 5 mg/kg × 4, 2 h apart (N = 54) each dose in a volume of 200 μL saline. ASN (Sigma-Aldrich, St. Luis, MO, USA) was dissolved in warmed (37 °C) saline and administered i.p. at the dose of either 500 or 1000 mg/kg × 4 (N = 72) in an injection volume of 300 μL. In combined ASN + METH treatments (N = 36), ASN was administered 30 min before each METH injection. Control mice (N = 18) received the vehicle (saline solution) in a volume of 200 μL.

Mice were divided in subgroups and they were sacrificed at different time intervals after treatments.

In detail, in order to evaluate how treatments affect the autophagy pathway focally within the mesencephalic tegmentum, where DA nuclei are hosted, mice were sacrificed at 48 h (N = 4/group) after the last injection. In these mice, brain slices corresponding to the midbrain tegmentum were used to analyze at confocal microscopy the expression of specific autophagy proteins (namely, Beclin1, LC3 and Cathepsin D) and the apoptotic marker Bax.

The remaining mice (N = 14/group) were sacrificed 7 days after treatments to assess neurotoxicity by morphological and biochemical analysis.

Biochemical assessment of nigro-striatal DA level was carried out by high-performance liquid chromatography (HPLC, N = 10/group). Immunohistochemical analysis of the catecholamine-synthesizing enzyme TH was carried out within striatal slices (N = 4/group).

Moreover, to investigate whether inhibition of autophagy extends METH-induced neurotoxicity to mesencephalic DA cell bodies, serial sections corresponding to the SNpc and VTA were collected and stained either with haematoxilin and eosin (H&E) or primary antibodies against TH. These slices were used to perform a stereology-like neuronal count [46], as reported in detail below. Other slices from SNpc and VTA were used for representative anti-GFAP immunohistochemistry.

### 4.3. HPLC Assay

The rostral striatum (N = 10 per experimental group) was dissected through the lateral ventricle and placed within an Eppendorf containing 0.6 mL of ice-cold 0.1 M perchloric acid (Sigma-Aldrich, St. Luis, MO, USA). After sonication, an aliquot of the homogenate (50 µL) was assayed for protein [85]. After centrifugation at 8000× *g* for 10 min, 20 µL of the clear supernatant was injected into an HPLC system where DA was analyzed as previously described [31], by using a reversed phase column (250 × 4.5 mm, C18, SGE) and two coulometric electrochemical detectors [31]. Reducing electrode was used for the quantitative analysis. The mobile phase consisted of a citrate-phosphate buffer (0.04 M citric acid (Sigma-Aldrich, St. Luis, MO, USA), 0.06 M Na_2_HPO_4_·2H_2_O) containing 0.1 mM EDTA, 0.6 mM 1-heptanesulphonic acid sodium salt and 10% methanol.

A standard curve was prepared using known amounts of DA (Sigma-Aldrich, St. Luis, MO, USA) dissolved in perchloric acid (0.1 M) containing a constant amount (10 pg/μL) of the internal standard (DBA; Sigma-Aldrich) and it was calculated using regression analysis of the ratios of the peak areas (DA area/DBA area) for various concentrations recorded at the reducing electrode. Values are given as the mean ± S.E.M. of values obtained in each experimental group.

### 4.4. Sample Preparation for Light and Confocal Microscopy

Mice (N = 8 per experimental group) were anaesthetized with chloral hydrate and then perfused trans-cardially with saline solution, followed by a fixing solution consisting of 4% paraformaldehyde (Sigma-Aldrich, St. Luis, MO, USA) in 0.1 M phosphate buffer, pH 7.3. Brain was dissected out and plunged into the same fixing solution for 24 h. Post-fixed brains were transferred in 70% ethylic alcohol overnight at 4 °C, dehydrated in increasing alcohol solutions, immersed in xylene for several hours and, finally, embedded in paraffin. Brains were sectioned coronally using a microtome in order to obtain 7 μm-thick slices, which were collected on polylysine-coated slides and were used for confocal and light microscopy, as described below.

### 4.5. Haematoxylin and Eosin

Sections were de-waxed by immersion in xylene for 40 min, they were re-hydrated in decreasing ethylic alcohol solutions and then stained with hematoxylin solution (cod# MHS32, Sigma-Aldrich) for a few minutes. Hematoxylin staining was stopped by washing in running water. Then, the sections were plunged into the eosin solution (cod# HT110216, Sigma-Aldrich, St. Luis, MO, USA). After repeated washing with distilled water to remove the excess of dye, sections were dehydrated in increasing ethylic alcohol solutions, clarified in xylene and finally, covered with DPX mounting medium (cod# 06522, Sigma-Aldrich, St. Luis, MO, USA). H&E-stained cells were observed under Nikon Eclipse 80i light microscope (Nikon, Tokyo, Japan), equipped with a digital camera connected to the NIS Elements software for image analysis (Nikon).

### 4.6. Immunofluorescence

Immunofluorescence was performed in de-waxed slices following our published protocol. Proteins of interest were revealed by subsequent incubation of the tissue with a primary (first step) and a secondary (second step) antibody. In the first step, the following primary antibodies were used: mouse monoclonal anti-Beclin-1 (cod# 612112, BD Biosciences, San Jose, CA, USA), rabbit polyclonal anti-LC3B (cod# L7543, Sigma-Aldrich, St. Luis, MO, USA), rabbit polyclonal anti-Bax (cod# 2772, Cell Signaling Technology Inc., Danvers, MA, USA) and rabbit anti-Cathepsin D (produced in our laboratory) (Laboratory of Dept of Health Sciences, Università del Piemonte Orientale, Novara, Italy). The following day, coverslips were incubated for 1 h at room temperature with secondary antibodies, either IRIS-2 (green fluorescence)- or IRIS-3 (red fluorescence)-conjugated Goat-anti rabbit IgG or Goat-anti mouse IgG (cod# 2W5-08, cod# 2w5-07, cod# 3w5-08, cod# 3w5-07; Cyanine Technology, Torino, Italy), as appropriate. Nuclei were stained with UV fluorescent dye DAPI (4′,6-diamidino-2-phenylindole, cod. 32670, Sigma-Aldrich, St. Luis, MO, USA).

The sections were washed with 0.1% Triton-PBS (Sigma-Aldrich, St. Luis, MO, USA) and mounted using SlowFade reagent (cod# S36936; Life Technologies, Paisley, UK). Images were captured with the confocal fluorescence microscope Leica DMIRE2 (Leica Microsystems AG, Wetzlad, Germany) equipped with Leica Confocal Software v. 2.61. Images shown are representative of at least three replicates.

Densitometric analysis of immunofluorescence (Int.DEN, integrated density) was performed with the software Image J. At least five microscopic fields randomly chosen were evaluated and the integrated density of fluorescence (i.e., the product of Area of selected cell X Mean fluorescence) was averaged and the data expressed as Arbitrary Units ± S.D.

Quantification of LC3 immunofluorescence was carried out by measuring LC3 immunopositive area per cell within five distinct microscopic fields following each specific treatment. Such an area was calculated by using Image J. In order to weight appropriately the immunofluorescent area to variability in cell size, the histochemistry-based cell area calculated with the same software on H&E-stained sections was used as a reference. This mean area corresponds to 278.0 ± 2.5 μm^2^. This calculation was carried out taking as a reference phenotype the pyramidal neurons of the ventral tier within SNpc. This area contains DA neurons, which are mainly susceptible to meso-striatal degeneration, which feature a pyramidal-like shape with a remarkable consistency of cell area. This made the neuronal population selected for measurement for LC3-immunofluorescent area very homogeneous (S.E.M. < 1% of the mean) concerning cell size.

### 4.7. Immunohistochemistry

Sections were de-waxed in xylene (Sigma-Aldrich, St. Luis, MO, USA), re-hydrated in decreasing concentrations of ethylic alcohol and after permeabilization in 0.01% Triton X-100, were immersed in 3% H_2_O_2_ to inhibit the endogenous peroxidases and then incubated in 10% normal goat serum for 2 h. Then, they were incubated overnight at 4 °C with the primary antibody solution containing 2% normal goat serum in TBS. The mouse anti-TH primary antibody (cod# T1299, Sigma-Aldrich) and the mouse anti-GFAP primary antibody (cod# G3893, Sigma-Aldrich) were diluted 1:1000. The antigen-antibody reaction was revealed using the anti-mouse biotin-conjugated secondary antibody (cod# BA9200, Vector Laboratories, Burlingame, CA, USA), diluted 1:200 for 1 h at room temperature (RT), followed by avidin-biotin complex (ABC, cod# PK6100, Vector Laboratories, Burlingame, CA, USA) for 1 h and the peroxidase substrate diaminobenzidine (DAB, cod# SK4100, Vector Laboratories) for 3 min at RT. After DAB reaction, slides bearing sections of SNpc and VTA were counterstained with the basic dye haematoxylin, which allowed to visualize all the cell population by identifying the cell nuclei. Finally, cells were dehydrated in increasing alcohol solutions. After washing in PBS and clarified in xylene, slices were cover-slipped with DPX mounting medium (cod# 06522, Sigma-Aldrich) and observed at light microscopy (Nikon).

Densitometric analysis related to TH or GFAP immunoperoxidase intensity was calculated with the software Image J. In detail, densitometry of TH immunostaining within the striatum was carried out within 5 sections/mouse. To measure densitometry of TH and GFAP immunostaining within the midbrain tegmentum, a square-shaped area (100 × 100 μm^2^), placed roughly at 0.8–1 mm laterally to the midline, was selected within 5 sections/mouse.

### 4.8. Stereology-like Neuronal Count

The stereology-like analysis of the SNpc and VTA neurons was carried out in midbrain sections of N = 4 mice per group following H&E and TH immunohistochemistry. Sections used for the cell count were selected following the criteria described in previous studies [46], which guarantee to correctly identify and quantify specific neurons within a selected brain area.

The exact identification of the rostro-caudal limits of the SNpc and VTA was carried out by referring to the Paxinos and Franklin atlas (2004) [86]. In particular, for the SNpc the sections analyzed were included within the AP stereotaxic coordinates, which extend from 2.70 mm to 3.80 mm posterior to bregma. These sections also comprehend the VTA, which, in fact, extends from 2.90 mm to 3.80 mm posterior to bregma.

Within these regions, nigral cells were identified by combining morphological and size-exclusion criteria [46]. Briefly, only cells exhibiting elongated or pyramidal cell bodies, euchromatic nuclei with evident nucleoli and ranging from about 30 μm up to 40 μm were included in the count [87].

In order to count the highest number of nigral neurons and avoid double counting of the same neurons, taking into account the average cell body diameter of nigral neurons in this mouse strain [87], consecutive sections (namely, one out of five), spaced at least 35 μm each other, were collected. These sections were alternatively stained for H&E and TH immunostaining and then used for cell count.

Within each TH-immunostained section, TH-non-ir cells intermingled with TH-ir cells were identified through their haematoxylin-counterstained nucleus and were counted separately. The number of TH-ir and TH-non-ir neurons counted in each section was plotted in a graph, along with the corresponding AP coordinates.

Counts were carried out at light microscopy (Nikon) at 20× magnification by an observer blind to the treatments.

Values were expressed as the mean ± S.E.M. of TH-ir cells counted at each AP level or as the mean ± S.E.M. of total TH-ir and TH-non-ir cells or total H&E-stained cells within the entire SNpc and VTA for each experimental group.

### 4.9. Statistical Analysis

All data per each group are given as the mean ± S.E.M. Comparisons between groups concerning biochemical analysis and stereology-like cell counts were carried out by One-way analysis of variance ANOVA, followed by Scheffè’s post-hoc analysis.

Statistical analysis of data related to immunofluorescence densitometry shown in Figure 1B was carried out by GraphPad Prism 5.0 software. Tukey’s multiple comparison test after one-way ANOVA analysis was employed. These data are given as the mean ± S.D.

Since the immunofluorescence in vivo is difficult to be counted as single monomorfic puncta, to quantify the expression of LC3 immunofluorescence the mean + S.E.M. of LC3 immunopositive area per cell was calculated following each specific treatment. Such an area was measured by using a software-based analysis (Image J). In order to weight appropriately the immunofluorescent area to potential variability in cell size, the histochemistry-based cell area calculated with the same software on H&E-stained sections was used as a reference. This calculation was carried out taking as a reference phenotype the pyramidal neurons of the ventral tier within SNpc. In fact, this specific area (278.0 ± 2.5 μm^2^) corresponds to the one where LC3 immunofluorescence was carried out as shown in representative pictures.

Differences between groups were considered statistically significant when the null hypothesis (H_0_) was *p* ≤ 0.05.

### 4.10. Experiments Related to GLN and METH Treatments

Materials and methodological procedures related to GLN and METH treatments are reported in detail within the Supplementary Materials.

## 5. Conclusions

The present data lead to hypothesize that, in vivo, DA toxicity for DA cell bodies is rare due to the persistence of effective autophagy as a defense mechanism within the neuronal cell bodies, which impedes METH to exert a neurotoxic effect. Such a process is less effective at the level of the axon terminals, where the autophagy machinery is massively challenged by the great amount of oxidative stress, which is produced by a massive DA concentration from disrupted neurotransmitter vesicles, which, by definition, does not take place in the cell body. In the present study, the natural refractoriness of the cell body to METH toxicity was removed by administering in vivo autophagy inhibitors such as ASN or GLN in combination with METH. ASN, along with GLN is a naturally occurring compound, which exerts a powerful inhibition of the autophagy machinery both inhibiting the autophagy induction and the autophagy flux [35,36,37,38,39,40,41,42,43,44,45].

Although slight discrepancies exist concerning the effects of ASN and GLN when administered alone or in combination with METH, a significant and robust reduction of LC3-immunofluorescent area was always measured. The slight numerical discrepancies between the effects of ASN and those of GLN, when both compounds are administered alone, are likely to be due to slight differences in the molecular mechanisms of ASN compared with GLN [35,36,37,38,39,40,41,42,43,44,45]. Nonetheless, the present data indicate that both compounds significantly and robustly inhibit the autophagy pathway during METH administration. This is evident both by measuring the LC3 immunofluorescent area per cell.

As shown in the mesencephalic tegmentum, when ASN is administered systemically, an impairment of the autophagy machinery is measured in situ within DA nuclei. In this condition, METH administration, at a dose, which by itself is not effective in producing nigral DA cell loss, becomes frankly toxicant to SNpc DA neurons and it extends toxicity to cell bodies within VTA. This is in line with recent findings showing that, mice deficient for the autophagy protein Atg7 (Atg7 knock-out mice) undergo spontaneous DA degeneration [88] that is counteracted by the autophagy activator rapamycin [89]. This extends findings indicating that rapamycin protects in vivo against MPTP toxicity [90] and in vitro against METH toxicity [30]. Again, the present findings are consistent with a number of reports showing that genetic parkinsonism is mostly linked to genes coding for proteins related to various steps of the autophagy pathway [89]. The remarkable effects produced by METH in the present experimental conditions at the level of the VTA deserve specific attention since this may implicate a potential deleterious effect of METH on cognition and mood. It is well-known that chronic METH abusers suffer of cognitive impairment, which persists after years of METH withdrawal [91,92,93,94,95]. The VTA has been recently posed as a key point in the genesis of dementia [1,96,97]. Thus, it is likely that a deficiency in the autophagy machinery, which, in fact, occurs in Alzheimer’s disease patients, may induce dementia not simply by altering cortical neurons but also by impairing DA cells, which in turn project to the cortex. Again, a recent paper by Weinshenker (2018) [98] indicates that similar phenomena may take place in norepinephrine neurons, due to metabolic alterations, which are similar to that produced by METH within DA neurons. DA metabolites such as 3,4-dihydroxyphenylacetaldehyde (DOPALD) may be toxic [99] as much as an analogous aldehyde, which is produced within norepinephrine neurons of the locus coeruleus (3,4-dihydroxyphenylglycol aldehyde, DOPEGALD) [98,100].

## Figures and Tables

**Table 1:** LC3 immunofluorescent areas within the ventral tier neurons of the SNpc.

	Controls	METH	ASN	ASN + METH
**Immunofluorescent area (μm^2^)/cell**	2.67 ± 0.27	62.31 ± 10.17 *	0.72 ± 0.03	23.47 ± 4.70 ^#^
*n*° Tot cells	26	24	25	20
	**Controls**	**METH**	**GLN**	**GLN + METH**
**Immunofluorescent area (μm^2^)/cell**	1.53 ± 0.30	78.14 ± 6.20 *	0.86 ± 0.21	19.68 ± 2.21 ^#^
*n*° Tot cells	25	24	24	20

Cell size was measured by Image J taking as a reference phenotype the pyramidal neurons of SNpc ventral tier. * *p* < 0.05 compared with controls. ^#^ *p* < 0.05 compared with METH.

## Data Availability

Data is contained within the article or Supplementary Materials.

## Supplementary Materials

The following are available online at https://www.mdpi.com/article/10.3390/ph14101003/s1, Figure S1: Densitometric analisys of TH and GFAP immunoperoxidase. Figure S2: GLN administration worsens METH-induced striatal loss of TH immunostaining. Figure S3: GLN extends METH toxicity to cell bodies within SNpc and VTA.

## References

[1] Fornai F., Puglisi-Allegra S. (2021). Autophagy status as a gateway for stress-induced catecholamine interplay in neurodegeneration. Neurosci. Biobehav. Rev..

[2] Hemmerle A.M., Herman J.P., Seroogy K.B. (2012). Stress, depression and Parkinson’s disease. Exp. Neurol..

[3] Austin K.W., Ameringer S.W., Cloud L.J. (2016). An Integrated Review of Psychological Stress in Parkinson’s Disease: Biological Mechanisms and Symptom and Health Outcomes. Parkinson’s Dis..

[4] Matuszewich L., Yamamoto B.K. (2004). Effects of chronic stress on methamphetamine-induced dopamine depletions in the striatum. Ann. N. Y. Acad. Sci..

[5] Quinton M.S., Yamamoto B.K. (2007). Neurotoxic effects of chronic restraint stress in the striatum of methamphetamine-exposed rats. Psychopharmacology.

[6] Wu Q., Yang X., Zhang Y., Zhang L., Feng L. (2016). Chronic mild stress accelerates the progression of Parkinson’s disease in A53T α-synuclein transgenic mice. Exp. Neurol..

[7] Gutierrez A., Jablonski S.A., Amos-Kroohs R.M., Barnes A.C., Williams M.T., Vorhees C.V. (2017). Effects of Housing on Methamphetamine-Induced Neurotoxicity and Spatial Learning and Memory. ACS Chem. Neurosci..

[8] O’Dell S.J., Weihmuller F.B., Marshall J.F. (1991). Multiple methamphetamine injections induce marked increases in extracellular striatal dopamine which correlate with subsequent neurotoxicity. Brain Res..

[9] Hutchison J.B., Strupish J., Nahorski S.R. (1986). Release of endogenous dopamine and cholecystokinin from rat striatal slices: Effects of amphetamine and dopamine antagonists. Brain Res..

[10] Hurd Y.L., Ungerstedt U. (1989). Cocaine: An in vivo microdialysis evaluation of its acute action on dopamine transmission in rat striatum. Synapse.

[11] Guix T., Hurd Y.L., Ungerstedt U. (1992). Amphetamine enhances extracellular concentrations of dopamine and acetylcholine in dorsolateral striatum and nucleus accumbens of freely moving rats. Neurosci. Lett..

[12] Miczek K.A., Mutschler N.H., van Erp A.M., Blank A.D., McInerney S.C. (1999). d-Amphetamine “cue” generalizes to social defeat stress: Behavioral sensitization and attenuated accumbens dopamine. Psychopharmacology.

[13] Marsteller D.A., Gerasimov M.R., Schiffer W.K., Geiger J.M., Barnett C.R., Schaich Borg J., Scott S., Ceccarelli J., Volkow N.D., Molina P.E. (2002). Acute handling stress modulates methylphenidate-induced catecholamine overflow in the medial prefrontal cortex. Neuropsychopharmacology.

[14] Antelman S.M., Eichler A.J., Black C.A., Kocan D. (1980). Interchangeability of stress and amphetamine in sensitization. Science.

[15] Larsen K.E., Fon E.A., Hastings T.G., Edwards R.H., Sulzer D. (2002). Methamphetamine-induced degeneration of dopaminergic neurons involves autophagy and upregulation of dopamine synthesis. J. Neurosci..

[16] Cabib S., Puglisi-Allegra S. (2012). The mesoaccumbens dopamine in coping with stress. Neurosci. Biobehav. Rev..

[17] Post R.M. (2016). Epigenetic basis of sensitization to stress, affective episodes, and stimulants: Implications for illness progression and prevention. Bipolar Disord..

[18] Douma E.H., de Kloet E.R. (2020). Stress-induced plasticity and functioning of ventral tegmental dopamine neurons. Neurosci. Biobehav. Rev..

[19] Xiao X., Shang X., Zhai B., Zhang H., Zhang T. (2018). Nicotine alleviates chronic stress-induced anxiety and depressive-like behavior and hippocampal neuropathology via regulating autophagy signaling. Neurochem. Int..

[20] Puri D., Subramanyam D. (2019). Stress-(self) eating: Epigenetic regulation of autophagy in response to psychological stress. FEBS J..

[21] Jung S., Choe S., Woo H., Jeong H., An H.K., Moon H., Ryu H.Y., Yeo B.K., Lee Y.W., Choi H. (2020). Autophagic death of neural stem cells mediates chronic stress-induced decline of adult hippocampal neurogenesis and cognitive deficits. Autophagy.

[22] Castino R., Lazzeri G., Lenzi P., Bellio N., Follo C., Ferrucci M., Fornai F., Isidoro C. (2008). Suppression of autophagy precipitates neuronal cell death following low doses of methamphetamine. J. Neurochem..

[23] Seiden L.S., Fischman M.W., Schuster C.R. (1976). Long-term methamphetamine induced changes in brain catecholamines in tolerant rhesus monkeys. Drug Alcohol Depend..

[24] Schmidt C.J., Ritter J.K., Sonsalla P.K., Hanson G.R., Gibb J.W. (1985). Role of dopamine in the neurotoxic effects of methamphetamine. J. Pharmacol. Exp. Ther..

[25] Sonsalla P.K., Heikkila R.E. (1988). Neurotoxic effects of 1-methyl-4-phenyl-1,2,3,6-tetrahydropyridine (MPTP) and methamphetamine in several strains of mice. Prog. Neuropsychopharmacol. Biol. Psychiatry.

[26] Wilson J.M., Kalasinsky K.S., Levey A.I., Bergeron C., Reiber G., Anthony R.M., Schmunk G.A., Shannak K., Haycock J.W., Kish S.J. (1996). Striatal dopamine nerve terminal markers in human, chronic methamphetamine users. Nat. Med..

[27] Volkow N.D., Chang L., Wang G.J., Fowler J.S., Leonido-Yee M., Franceschi D., Sedler M.J., Gatley S.J., Hitzemann R., Ding Y.S. (2001). Association of dopamine transporter reduction with psychomotor impairment in methamphetamine abusers. Am. J. Psychiatry.

[28] Cubells J.F., Rayport S., Rajendran G., Sulzer D. (1994). Methamphetamine neurotoxicity involves vacuolation of endocytic organelles and dopamine-dependent intracellular oxidative stress. J. Neurosci..

[29] Fornai F., Lenzi P., Gesi M., Soldani P., Ferrucci M., Lazzeri G., Capobianco L., Battaglia G., De Blasi A., Nicoletti F. (2004). Methamphetamine produces neuronal inclusions in the nigrostriatal system and in PC12 cells. J. Neurochem..

[30] Lazzeri G., Biagioni F., Fulceri F., Busceti C.L., Scavuzzo M.C., Ippolito C., Salvetti A., Lenzi P., Fornai F. (2018). mTOR Modulates Methamphetamine-Induced Toxicity through Cell Clearing Systems. Oxid. Med. Cell Longev..

[31] Lazzeri G., Lenzi P., Busceti C.L., Ferrucci M., Falleni A., Bruno V., Paparelli A., Fornai F. (2007). Mechanisms involved in the formation of dopamine-induced intracellular bodies within striatal neurons. J. Neurochem..

[32] Hastings T.G., Zigmond M.J. (1994). Identification of catechol-protein conjugates in neostriatal slices incubated with [3H]dopamine: Impact of ascorbic acid and glutathione. J. Neurochem..

[33] Hastings T.G., Lewis D.A., Zigmond M.J. (1996). Role of oxidation in the neurotoxic effects of intrastriatal dopamine injections. Proc. Natl. Acad. Sci. USA.

[34] LaVoie M.J., Hastings T.G. (1999). Dopamine quinone formation and protein modification associated with the striatal neurotoxicity of methamphetamine: Evidence against a role for extracellular dopamine. J. Neurosci..

[35] Seglen P.O., Gordon P.B., Poli A. (1980). Amino acid inhibition of the autophagic/lysosomal pathway of protein degradation in isolated rat hepatocytes. Biochim. Biophys. Acta.

[36] Seglen P.O., Gordon P.B., Grinde B., Solheim A., Kovács A.L., Poli A. (1981). Inhibitors and pathways of hepatocytic protein degradation. Acta Biol. Med. Ger..

[37] Kopitz J., Kisen G.O., Gordon P.B., Bohley P., Seglen P.O. (1990). Nonselective autophagy of cytosolic enzymes by isolated rat hepatocytes. J. Cell Biol..

[38] Høyvik H., Gordon P.B., Berg T.O., Strømhaug P.E., Seglen P.O. (1991). Inhibition of autophagic-lysosomal delivery and autophagic lactolysis by asparagine. J. Cell Biol..

[39] Gordon P.B., Høyvik H., Seglen P.O. (1992). Prelysosomal and lysosomal connections between autophagy and endocytosis. Biochem. J..

[40] Fengsrud M., Roos N., Berg T., Liou W., Slot J.W., Seglen P.O. (1995). Ultrastructural and immunocytochemical characterization of autophagic vacuoles in isolated hepatocytes: Effects of vinblastine and asparagine on vacuole distributions. Exp. Cell Res..

[41] Meng D., Yang Q., Wang H., Melick C.H., Navlani R., Frank A.R., Jewell J.L. (2020). Glutamine and asparagine activate mTORC1 inde-pendently of Rag GTPases. J. Biol. Chem..

[42] Nicklin P., Bergman P., Zhang B., Triantafellow E., Wang H., Nyfeler B., Yang H., Hild M., Kung C., Wilson C. (2009). Bidirectional transport of amino acids regulates mTOR and autophagy. Cell.

[43] Durán R.V., Oppliger W., Robitaille A.M., Heiserich L., Skendaj R., Gottlieb E., Hall M.N. (2012). Glutaminolysis activates Rag-mTORC1 signaling. Mol. Cell.

[44] Kim S.G., Hoffman G.R., Poulogiannis G., Buel G.R., Jang Y.J., Lee K.W., Kim B.Y., Erikson R.L., Cantley L.C., Choo A.Y. (2013). Metabolic stress controls mTORC1 lysosomal localization and dimerization by regulating the TTT-RUVBL1/2 complex. Mol. Cell.

[45] Jewell J.L., Kim Y.C., Russell R.C., Yu F.X., Park H.W., Plouffe S.W., Tagliabracci V.S., Guan K.L. (2015). Metabolism. Differential regulation of mTORC1 by leucine and glutamine. Science.

[46] Ferrucci M., Lazzeri G., Flaibani M., Biagioni F., Cantini F., Madonna M., Bucci D., Limanaqi F., Soldani P., Fornai F. (2018). In search for a gold-standard procedure to count motor neurons in the spinal cord. Histol. Histopathol..

[47] Weihmuller F.B., O’Dell S.J., Marshall J.F. (1992). MK-801 protection against methamphetamine-induced striatal dopamine terminal injury is associated with attenuated dopamine overflow. Synapse.

[48] Eisch A.J., Gaffney M., Weihmuller F.B., O’Dell S.J., Marshall J.F. (1992). Striatal subregions are differentially vulnerable to the neurotoxic effects of methamphetamine. Brain Res..

[49] Baumann M.H., Ayestas M.A., Sharpe L.G., Lewis D.B., Rice K.C., Rothman R.B. (2002). Persistent antagonism of methamphetamine-induced dopamine release in rats pretreated with GBR12909 decanoate. J. Pharmacol. Exp. Ther..

[50] Riddle E.L., Fleckenstein A.E., Hanson G.R. (2006). Mechanisms of methamphetamine-induced dopaminergic neurotoxicity. AAPS J..

[51] Cadet J.L., Ali S., Epstein C. (1994). Involvement of oxygen-based radicals in methamphetamine-induced neurotoxicity: Evidence from the use of CuZnSOD transgenic mice. Ann. N. Y. Acad. Sci..

[52] Miyazaki I., Asanuma M., Diaz-Corrales F.J., Fukuda M., Kitaichi K., Miyoshi K., Ogawa N. (2006). Methamphetamine-induced dopaminergic neurotoxicity is regulated by quinone-formation-related molecules. FASEB J..

[53] Sulzer D. (2011). How addictive drugs disrupt presynaptic dopamine neurotransmission. Neuron.

[54] Ingram S.L., Prasad B.M., Amara S.G. (2002). Dopamine transporter-mediated conductances increase excitability of midbrain dopamine neurons. Nat. Neurosci..

[55] John C.E., Jones S.R. (2007). Voltammetric characterization of the effect of monoamine uptake inhibitors and releasers on dopamine and serotonin uptake in mouse caudate-putamen and substantia nigra slices. Neuropharmacology.

[56] Yee A.G., Forbes B., Cheung P.Y., Martini A., Burrell M.H., Freestone P.S., Lipski J. (2019). Action potential and calcium dependence of tonic somatodendritic dopamine release in the Substantia Nigra pars compacta. J. Neurochem..

[57] Sonsalla P.K., Jochnowitz N.D., Zeevalk G.D., Oostveen J.A., Hall E.D. (1996). Treatment of mice with methamphetamine produces cell loss in the substantia nigra. Brain Res..

[58] Ladenheim B., Krasnova I.N., Deng X., Oyler J.M., Polettini A., Moran T.H., Huestis M.A., Cadet J.L. (2000). Methamphetamine-induced neurotoxicity is attenuated in transgenic mice with a null mutation for interleukin-6. Mol. Pharmacol..

[59] Ares-Santos S., Granado N., Espadas I., Martinez-Murillo R., Moratalla R. (2014). Methamphetamine causes degeneration of dopamine cell bodies and terminals of the nigrostriatal pathway evidenced by silver staining. Neuropsychopharmacology.

[60] Seiden L.S. (1985). Methamphetamine: Toxicity to dopaminergic neurons. NIDA Res. Monogr..

[61] Kataoka Y., Gomita Y., Fukuda T., Eto K., Araki Y. (1986). Effects of aggregation on methamphetamine toxicity in mice. Acta Med..

[62] Cragg S.J., Greenfield S.A. (1997). Differential autoreceptor control of somatodendritic and axon terminal dopamine release in substantia nigra, ventral tegmental area, and striatum. J. Neurosci..

[63] Kogan F.J., Nichols W.K., Gibb J.W. (1976). Influence of methamphetamine on nigral and striatal tyrosine hydroxylase activity and on striatal dopamine levels. Eur. J. Pharmacol..

[64] Wagner G.C., Ricaurte G.A., Seiden L.S., Schuster C.R., Miller R.J., Westley J. (1980). Long-lasting depletions of striatal dopamine and loss of dopamine uptake sites following repeated administration of methamphetamine. Brain Res..

[65] Fleckenstein A.E., Metzger R.R., Wilkins D.G., Gibb J.W., Hanson G.R. (1997). Rapid and reversible effects of methamphetamine on dopamine transporters. J. Pharmacol. Exp. Ther..

[66] Fumagalli F., Gainetdinov R.R., Valenzano K.J., Caron M.G. (1998). Role of dopamine transporter in methamphetamine-induced neurotoxicity: Evidence from mice lacking the transporter. J. Neurosci..

[67] Fleckenstein A.E., Volz T.J., Riddle E.L., Gibb J.W., Hanson G.R. (2007). New insights into the mechanism of action of amphetamines. Annu. Rev. Pharmacol. Toxicol..

[68] Volz T.J., Hanson G.R., Fleckenstein A.E. (2007). The role of the plasmalemmal dopamine and vesicular monoamine transporters in methamphetamine-induced dopaminergic deficits. J. Neurochem..

[69] Kokoshka J.M., Vaughan R.A., Hanson G.R., Fleckenstein A.E. (1998). Nature of methamphetamine-induced rapid and reversible changes in dopamine transporters. Eur. J. Pharmacol..

[70] Brown J.M., Hanson G.R., Fleckenstein A.E. (2000). Methamphetamine rapidly decreases vesicular dopamine uptake. J. Neurochem..

[71] Riddle E.L., Topham M.K., Haycock J.W., Hanson G.R., Fleckenstein A.E. (2002). Differential traffi cking of the vesicular monoamine transporter-2 by methamphetamine and cocaine. Eur. J. Pharmacol..

[72] Sandoval V., Riddle E.L., Hanson G.R., Fleckenstein A.E. (2002). Methylphenidate redistributes vesicular monoamine transporter-2: Role of dopamine receptors. J. Neurosci..

[73] Sandoval V., Riddle E.L., Hanson G.R., Fleckenstein A.E. (2003). Methylphenidate alters vesicular monoamine transport and prevents methamphetamine-induced dopaminergic deficits. J. Pharmacol. Exp. Ther..

[74] Sulzer D., Rayport S. (1990). Amphetamine and other psychostimulants reduce pH gradients in midbrain dopaminergic neurons and chromaffin granules: A mechanism of action. Neuron.

[75] Sulzer D., Pothos E., Sung H.M., Maidment N.T., Hoebel B.G., Rayport S. (1992). Weak base model of amphetamine action. Ann. N. Y. Acad. Sci..

[76] Green A.L., El Hait M.A. (1980). *p*-Methoxyamphetamine, a potent reversible inhibitor of type-A monoamine oxidase in vitro and in vivo. J. Pharm. Pharmacol..

[77] Suzuki O., Hattori H., Asano M., Oya M., Katsumata Y. (1980). Inhibition of monoamine oxidase by d-methamphetamine. Biochem. Pharmacol..

[78] Graham D.G., Tiffany S.M., Vogel F.S. (1978). The toxicity of melanin precursors. J. Invest. Dermatol..

[79] Cohen G. (1984). Oxy-radical toxicity in catecholamine neurons. Neurotoxicology.

[80] Schlüter O.M., Fornai F., Alessandrí M.G., Takamori S., Geppert M., Jahn R., Südhof T.C. (2003). Role of alpha-synuclein in 1-methyl-4-phenyl-1,2,3,6-tetrahydropyridine-induced parkinsonism in mice. Neuroscience.

[81] Cabib S. (1993). Strain-dependent behavioural sensitization to amphetamine: Role of environmental influences. Behav. Pharmacol..

[82] Kita T., Paku S., Takahashi M., Kubo K., Wagner G.C., Nakashima T. (1998). Methamphetamine-induced neurotoxicity in BALB/c, DBA/2N and C57BL/6N mice. Neuropharmacology.

[83] Fornai F. Experimental Models in Parkinson’s Disease. Proceedings of the LIMPE Seminars.

[84] Ferrucci M., Castino R., Lazzeri G., Cantafora E., Lenzi P., Isidoro C., Longone P., Paparelli A., Fornai F.E. (2008). Impairment of autophagy removes the resistance of dopaminergic cell bodies to methamphetamine toxicity. Session: Neurotoxicity of Amphetamine and Related Addictive Drugs.

[85] Lowry O.H., Rosembrough N.J., Farr A.L., Randall R.J. (1951). Protein measurement with the Folin phenol reagent. J. Biol. Chem..

[86] Paxinos G., Franklin K.B.J. (2004). The Mouse Brain in Stereotaxic Coordinates.

[87] Vidyadhara D.J., Yarreiphang H., Raju T.R., Alladi P.A. (2017). Admixing of MPTP-Resistant and Susceptible Mice Strains Augments Nigrostriatal Neuronal Correlates to Resist MPTP-Induced Neurodegeneration. Mol. Neurobiol..

[88] Friedman L.G., Lachenmayer M.L., Wang J., He L., Poulose S.M., Komatsu M., Holstein G.R., Yue Z. (2012). Disrupted autophagy leads to dopaminergic axon and dendrite degeneration and promotes presynaptic accumulation of α-synuclein and LRRK2 in the brain. J. Neurosci..

[89] Lu J., Wu M., Yue Z. (2020). Autophagy and Parkinson’s Disease. Adv. Exp. Med. Biol..

[90] Malagelada C., Jin Z.H., Jackson-Lewis V., Przedborski S., Greene L.A. (2010). Rapamycin protects against neuron death in in vitro and in vivo models of Parkinson’s disease. J. Neurosci..

[91] Wood S., Sage J.R., Shuman T., Anagnostaras S.G. (2013). Psychostimulants and cognition: A continuum of behavioral and cognitive activation. Pharmacol. Rev..

[92] Chen C.K., Lin S.K., Chen Y.C., Huang M.C., Chen T.T., Ree S.C., Wang L.J. (2015). Persistence of psychotic symptoms as an indicator of cognitive impairment in methamphetamine users. Drug Alcohol Depend..

[93] London E.D., Kohno M., Morales A.M., Ballard M.E. (2015). Chronic methamphetamine abuse and corticostriatal deficits revealed by neuroimaging. Brain Res..

[94] Potvin S., Pelletier J., Grot S., Hébert C., Barr A.M., Lecomte T. (2018). Cognitive deficits in individuals with methamphetamine use disorder: A meta-analysis. Addict. Behav..

[95] Proebstl L., Krause D., Kamp F., Hager L., Manz K., Schacht-Jablonowsky M., Straif M., Riebschläger M., Neumann S., Schreiber A. (2019). Methamphetamine withdrawal and the restoration of cognitive functions—A study over a course of 6 months abstinence. Psychiatry Res..

[96] D’Amelio M., Serra L., Bozzali M. (2018). Ventral Tegmental Area in Prodromal Alzheimer’s Disease: Bridging the Gap between Mice and Humans. J. Alzheimer’s Dis..

[97] Serra L., D’Amelio M., Di Domenico C., Dipasquale O., Marra C., Mercuri N.B., Caltagirone C., Cercignani M., Bozzali M. (2018). In vivo mapping of brainstem nuclei functional connectivity disruption in Alzheimer’s disease. Neurobiol. Aging.

[98] Weinshenker D. (2018). Long Road to Ruin: Noradrenergic Dysfunction in Neurodegenerative Disease. Trends Neurosci..

[99] Vecchio L.M., Sullivan P., Dunn A.R., Bermejo M.K., Fu R., Masoud S.T., Gregersen E., Urs N.M., Nazari R., Jensen P.H. (2021). Enhanced tyrosine hydroxylase activity induces oxidative stress, causes accumulation of autotoxic catecholamine metabolites, and augments amphetamine effects in vivo. J. Neurochem..

[100] Kang S.S., Liu X., Ahn E.H., Xiang J., Manfredsson F.P., Yang X., Luo H.R., Liles L.C., Weinshenker D., Ye K. (2020). Norepinephrine metabolite DOPEGAL activates AEP and pathological Tau aggregation in locus coeruleus. J. Clin. Investig..

