# Supplementary material for: Inhibition of Autophagy In Vivo Extends Methamphetamine Toxicity to Mesencephalic Cell Bodies
Source: Pharmaceuticals (Basel). 2021 Sep 29;14(10):1003. doi: 10.3390/ph14101003 (PMC8538796; doi:10.3390/ph14101003)
Supplement: Supplementary file 1 [file pharmaceuticals-14-01003-s001.zip › pharmaceuticals-1347752-supplementary.pdf]

Supplementary Figures

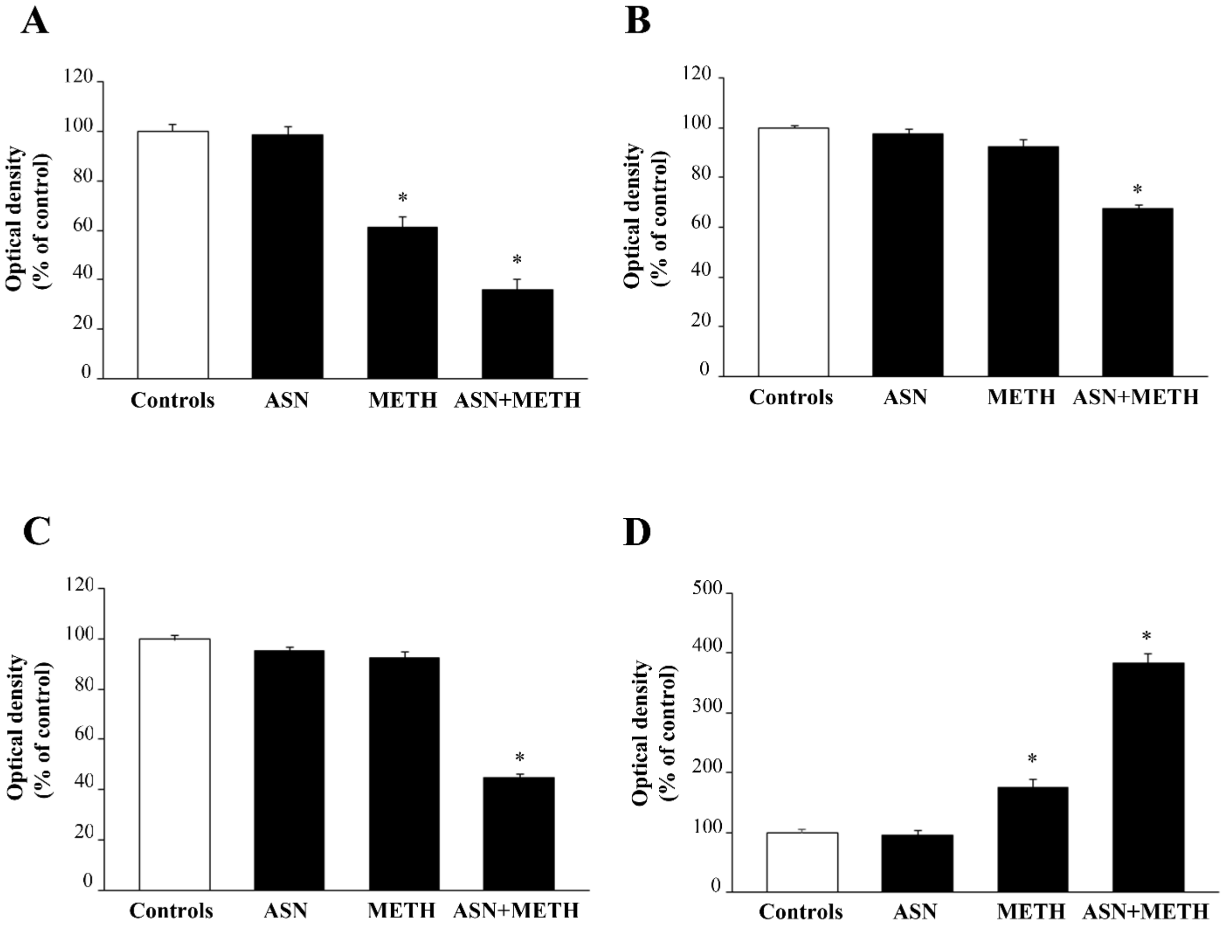

Supplementary Figure S1. Densitometric analisys of TH and GFAP immunoperoxidase. The graph report the densitometric analysis of TH immunoperoxidase measured in striatum (**A**), SNpc (**B**) and VTA (**C**). In (**D**) the densitometric analysis of GFAP immunoperoxidase within mesencephalic tegmentum is reported.
*P<0.05 compared with all other groups.

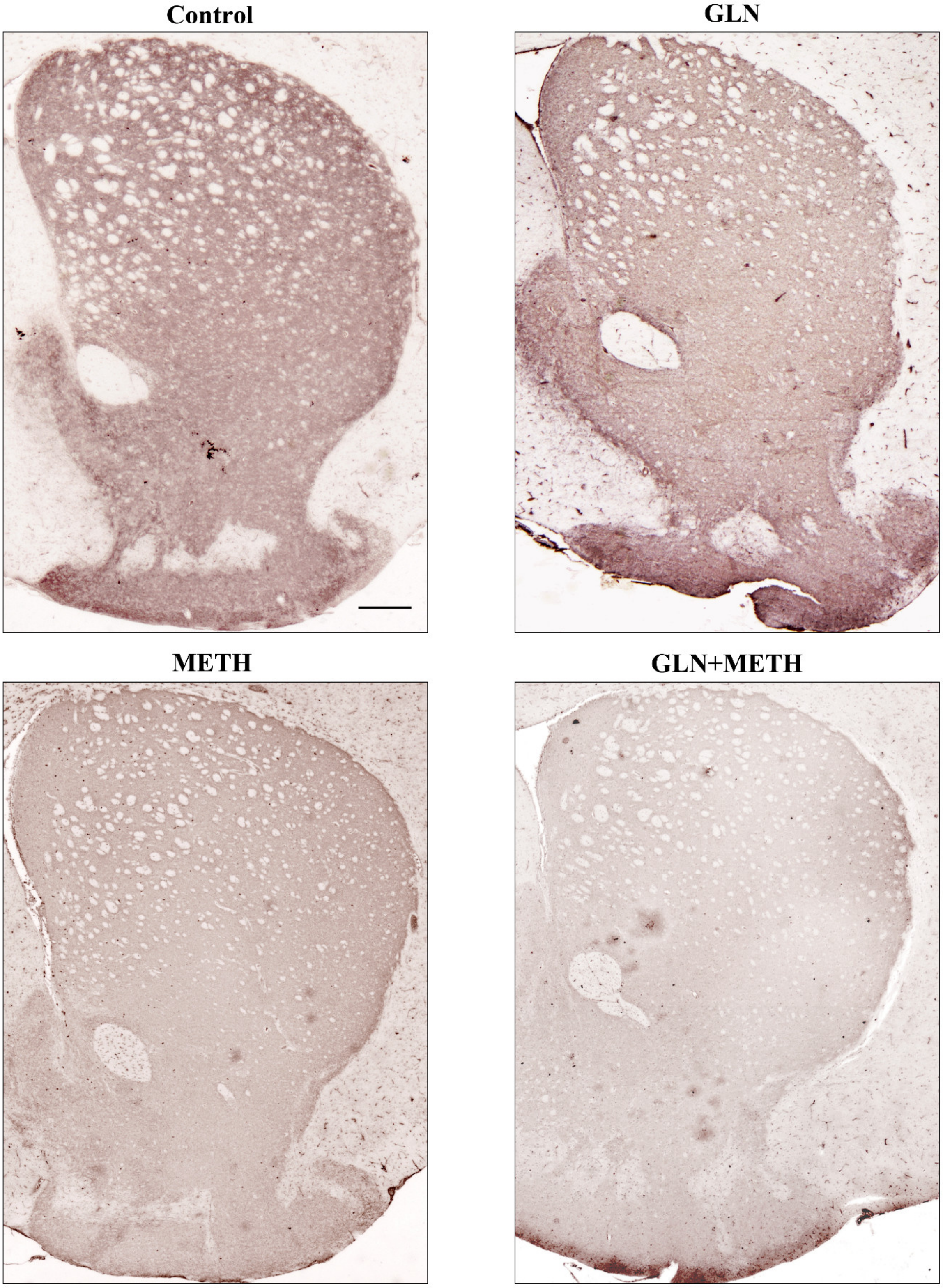

Supplementary Figure S2. GLN administration worsens METH-induced striatal loss of TH immunostaining. Representative pictures of TH immunohistochemistry within the striatum in a control mouse, and a mouse administered either GLN (1000 mg/kg x 4), or METH (5 mg/kg x 4), or combined GLN (1000 mg/kg x 4)+METH (5 mg/kg x 4).
Scale bar=340 μm.

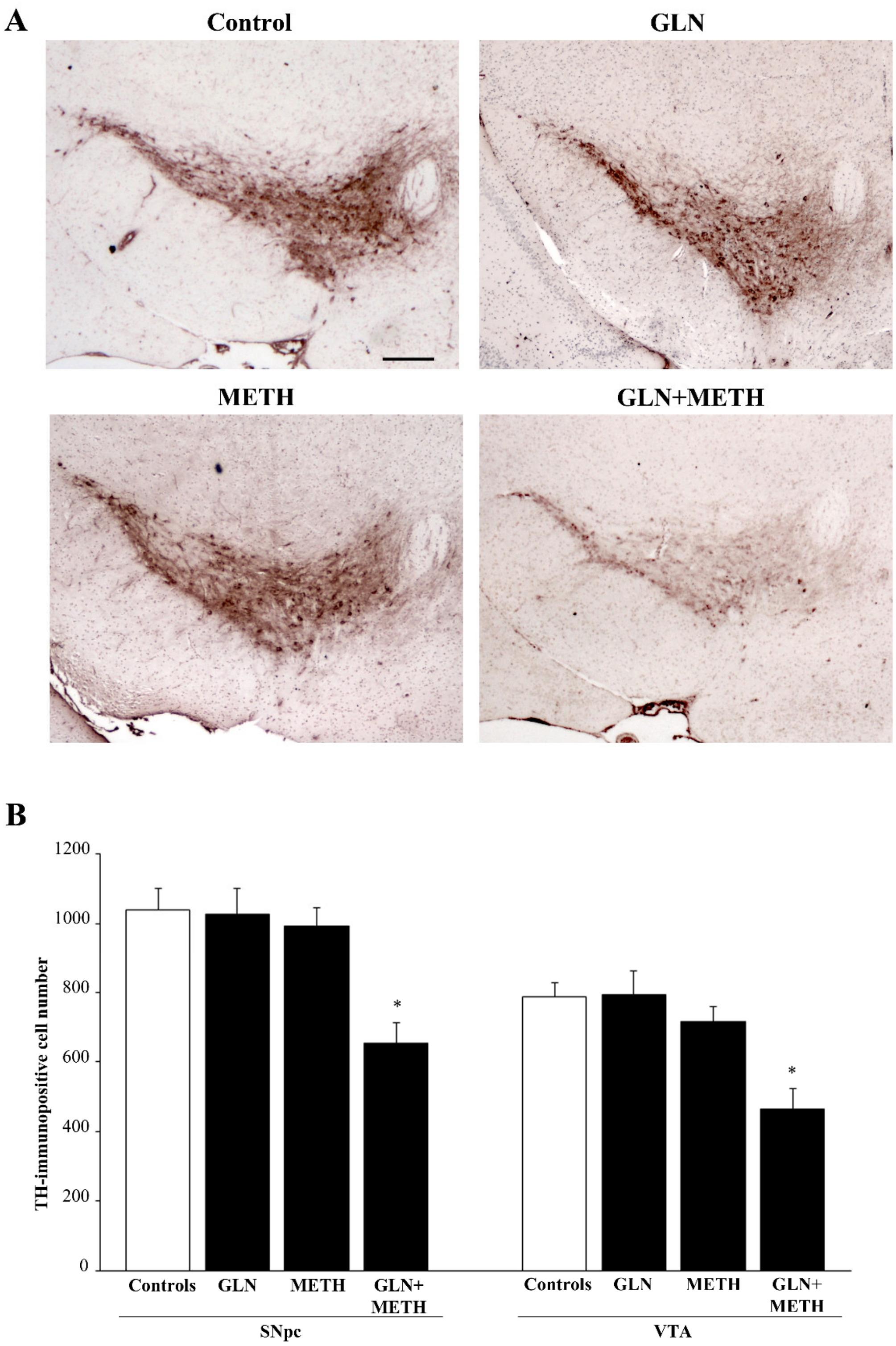

Supplementary Figure S3. GLN extends METH toxicity to cell bodies within SNpc and VTA.
(**A**) Representative pictures of TH immunostaining within the midbrain from a control mouse and from mice treated with GLN (1000 mg/kg x 4), or METH (5 mg/kg x 4), or combined GLN (1000 mg/kg x 4)+METH (5 mg/kg x 4). The count of TH-immunopositive cells within SNpc and VTA (**B**) was carried out in 4 sections per mouse following various treatments.
*P<0.05 compared with all other groups.
Scale bar=115 μm.

Supplementary Materials and Methods

1. Animals

C57Bl/6 male mice (N=32, Charles River Calco, Milano, Italy) were housed in small cages (N=3 per cage; cage length=25 cm; cage width=20 cm; cage height=13 cm) and kept under controlled environmental conditions (temperature=22 °C; humidity=40%; 12 hours light/ dark cycle) with food and water ad libitum. All these measures were kept constant all over the study since the effects of METH markedly vary depending on housing conditions.

Experiments were carried out according to the Guidelines of the European Council (86/609/EEC), and the NIH guide for the use and care of Experimental Animals. The experiments were approved by the local Ethical Committee.

2. Treatments

METH hydro-chloride (gently gifted by Medicina Legale, Forensic Medicine Institute, at the University of Pisa, Professor Mario Giusiani) was dissolved in saline and it was administered i.p. at the dose of 5 mg/kg x 4, 2 hours apart (N=16) each dose in a volume of 200 μL saline. Glutamine (GLN, Sigma-Aldrich, St. Luis, MO, USA) was dissolved in warmed (37 °C) saline and administered i.p. at the dose of 1000 mg/kg x 4 (N=16) in an injection volume of 300 μL. In combined GLN+METH treatments (N=8), ASN was administered 30 min before each METH injection. Control mice (N=8) received 200 μL of the vehicle (saline solution).

Mice were divided in two subgroups. The first subgroup (N=16) was sacrificed at 48 hours after the last METH injection to investigate the effects of the treatments on the autophagy pathway. In these mice the expression of specific autophagy proteins (namely, Beclin1, LC3, and Cathepsin D) and the apoptotic marker Bax within the mesencephalic tegmentum was evaluated by immunofluorescence. The second subgroup of mice (N=16) was sacrificed 7 days after the last METH injection and used to evaluate the effects of the treatments on the integrity of the meso-striatal system by analyzing the expression of the rate-limiting catecholamine-synthesizing enzyme tyrosine hydroxylase within striatum and midbrain tegmentum at light microscopy.

3. Sample preparation

Mice were anaesthetized with chloral hydrate and then transcardially perfused with saline solution, followed by a fixing solution consisting of 4% paraformaldehyde in 0.1 M phosphate buffer, pH 7.3. Brain was dissected out and plunged into the same fixing solution for 24 hours. Then brains were transferred in 70% ethylic alcohol overnight at 4 °C, dehydrated in increasing alcohol solutions, immersed in xylene for several hours, and finally embedded in paraffin. Brains were sectioned coronally using a microtome in order to obtain 7 μm-thick slices, which were collected on polylysine-coated slides.

4. Immunofluorescence

Sections corresponding to mesencephalic tegmentum were de-waxed in xylene, re-hydrated in decreasing concentrations of ethylic alcohol, and permeabilized in 0.01% Triton X-100. Then, slides were incubated in 10% normal goat serum for 2 hours, followed by solutions containing different primary antibodies diluted 1:100 and 2% NGS in TBS overnight at 4°C. The following primary antibodies were used: mouse anti-Cathepsin D (Sigma-Aldrich, cod# K4886), rabbit anti-LC3 (Abcam Cambridge, UK, cod# Ab128025), mouse anti-Beclin1 (Abcam, cod# Ab118148), and mouse anti-Bax (Santa Cruz Biotechnology, Dallas, TX, USA, cod# Sc-7480).

Afterwards, slides were washed in TBS and incubated for 1 hour with the anti-rabbit fluorophore-conjugated secondary antibodies (AlexaFluor 546, Life Technologies, Carlsbad, CA, USA, cod# A11010) diluted 1:200 or the anti-mouse fluorophore-conjugated secondary antibodies (AlexaFluor 488; Life Technologies, cod# A11001) diluted 1:200 in TBS at room temperature. After washing in TBS, slides were incubated with DAPI (Sigma-Aldrich, cod# D9664) diluted 1:1000 for 5 minutes to stain cell nuclei. Finally, slides were dehydrated using increasing alcohol solutions, clarified in xylene and covered with the mounting medium Fluoroshield (Sigma-Aldrich). Section were observed under fluorescence microscopy (Nikon). Merging of DAPI and different primary antibody fluorescence was obtained through NIS Elements software (Nikon).
Quantification of LC3 immunofluorescence was carried out by measuring LC3 immunopositive area per cell within five distinct microscopic fields following each specific treatment. Such an area was calculated by using Image J. In order to weight appropriately the immunofluorescent area to variability in cell size, the histochemistry-based cell area calculated with the same software on H&E-stained sections was used as a reference. This mean area corresponds to 278.0±2.5 μm^2. This calculation was carried out taking as a ref-erence phenotype the pyramidal neurons of the ventral tier within SNpc. This area con-tains DA neurons, which are mainly susceptible to meso-striatal degeneration, which feature a pyramidal-like shape with a remarkable consistency of cell area, which makes this specific population quite homogeneous concerning cell size.

5. Immunohistochemistry

Sections corresponding to striatum and mesencephalic tegmentum were de-waxed in xylene, re-hydrated in decreasing concentrations of ethylic alcohol, and after permeabilization in 0.01% Triton X-100 were immersed in 3% hydrogen peroxide to inhibit the endogenous peroxidases and incubated in 10% normal goat serum for 2 hours. Then, they were incubated overnight at 4 °C with a TBS solution containing the mouse anti-TH primary antibody (cod# T1299, Sigma-Aldrich) diluted 1:1000 and 2% normal goat serum. The antigen-antibody reaction was revealed using the anti-mouse biotin-conjugated secondary antibody (cod# BA9200, Vector Laboratories, Burlingame, CA, USA), diluted 1:200 for 1 hour at room temperature (RT), followed by avidin-biotin complex (ABC, cod# PK6100, Vector Laboratories) for 1 hour and the peroxidase substrate diaminobenzidine (DAB, cod# SK4100, Vector Laboratories) for 3 min at RT. Finally, cells were dehydrated in increasing alcohol solutions. After washing in PBS and clarified in xylene, slices were cover-slipped with DPX mounting medium (cod# 06522, Sigma-Aldrich) and observed at light microscopy (Nikon).

6. Neuronal count

Neuronal count of the SNpc and VTA neurons was carried out in selected slides (N=4/mouse) corresponding to the midbrain following TH immunohistochemistry. Sections used for the cell count were selected to count TH-immunopositive neurons at very same rostro-caudal level in each mouse. Briefly, starting from 3 mm posterior to the bregma, for each mouse four consecutive sections, at a distance of 210 μm (one out of 30), were collected (the last section corresponded roughly at 3.630 mm posterior to the bregma). The exact identification of the midbrain rostro-caudal levels was carried out by referring to the Paxinos and Franklin atlas (2004) [99].
Within these slides, TH-immunopositive cells were counted separately within SNpc and VTA.
Counts were carried out at light microscopy (Nikon) at 20× magnification by an observer blind to the treatments.
Values are given as the mean ± S.E.M. of the total TH-immunopositive cells counted within the selected SNpc and VTA slides for each experimental group.

7. **Statistical analysis**

Data related to the number of TH-immunopositive neurons within SNpc and VTA as well as LC3-immunofluorescent area/cell are given as the mean ± S.E.M. for each experimental group. In order to weight appropriately the immunofluorescence area to variability in cell size, the histochemistry-based cell area calculated with the same software on H&E-stained sections was used as a reference. This calculation was carried out taking as a reference phenotype the pyramidal neurons of the ventral tier within SNpc. In fact, this specific area ($278.0 \pm 2.5\ \mu m^2$) corresponds to the one where LC3 immunofluorescence was carried out as shown in representative pictures.

Comparisons between groups were carried out by One-way analysis of variance ANOVA, followed by Scheffè's post-hoc analysis. Differences between groups were considered statistically significant when the null hypothesis (H0) was $P \leq 0.05$.
